# The Role of E3 Ubiquitin Ligases and Deubiquitinases in Inflammatory Bowel Disease: Friend or Foe?

**DOI:** 10.3389/fimmu.2021.769167

**Published:** 2021-12-08

**Authors:** Min Zou, Qi-Shan Zeng, Jiao Nie, Jia-Hui Yang, Zhen-Yi Luo, Hua-Tian Gan

**Affiliations:** ^1^ Department of Gastroenterology and the Center of Inflammatory Bowel Disease, West China Hospital, Sichuan University, Chengdu, China; ^2^ Lab of Inflammatory Bowel Disease, Clinical Institute of Inflammation and Immunology, Frontiers Science Center for Disease-related Molecular Network, West China Hospital, Sichuan University, Chengdu, China; ^3^ Department of Geriatrics and National Clinical Research Center for Geriatric, West China Hospital, Sichuan University, Chengdu, China

**Keywords:** inflammatory bowel disease, E3 ubiquitin ligases, deubiquitinases, ubiquitination, deubiquitination

## Abstract

Inflammatory bowel disease (IBD), which include Crohn’s disease (CD) and ulcerative colitis (UC), exhibits a complex multifactorial pathogenesis involving genetic susceptibility, imbalance of gut microbiota, mucosal immune disorder and environmental factors. Recent studies reported associations between ubiquitination and deubiquitination and the occurrence and development of inflammatory bowel disease. Ubiquitination modification, one of the most important types of post-translational modifications, is a multi-step enzymatic process involved in the regulation of various physiological processes of cells, including cell cycle progression, cell differentiation, apoptosis, and innate and adaptive immune responses. Alterations in ubiquitination and deubiquitination can lead to various diseases, including IBD. Here, we review the role of E3 ubiquitin ligases and deubiquitinases (DUBs) and their mediated ubiquitination and deubiquitination modifications in the pathogenesis of IBD. We highlight the importance of this type of posttranslational modification in the development of inflammation, and provide guidance for the future development of targeted therapeutics in IBD.

## 1 Introduction

### 1.1 General Introduction of IBD

Inflammatory bowel disease (IBD) constitutes a group of chronic non-specific intestinal inflammatory disease which includes Crohn’s disease (CD) and ulcerative colitis (UC). CD can occur in any part of the digestive tract. Most of the lesions in CD are discontinuous changes that reach to the muscular layer although the mucosa between the lesions can appear completely normal. Histological findings of non-caseous granulomas are typically observed in patients with CD (1). UC lesions are mostly located in the colon and rectum, mainly in mucosa and submucosa, and show continuous and diffuse distribution. UC is histologically characterized by cryptitis or crypt abscess (2). The conventional therapeutic drugs for IBD include amino salicylic acid, glucocorticoids, immunosuppressants (azathioprine, cyclosporine), and biological agents such as infliximab, adalimumab, vedolizumab, and others (3). However, so far, there are no curative drugs or methods for IBD.

The global incidence of IBD has been increasing every year (4). Although the incidence in the United States and Europe appears to have stabilized, it is estimated that the number of patients in these two regions will reach 2.5 million and 3 million by 2030, respectively (5, 6). The incidence of IBD in developing countries is accelerating and will cause serious economic pressure and medical burden on patients, their families, and the society (7). Indeed, IBD has become a major social health problem in need of urgent action and novel treatments (5). To identify new therapeutic targets for IBD, researchers must first better understand the molecular mechanism underlying the pathology of this condition.

The exact etiology and pathogenesis of IBD remain unclear, although it is generally believed that IBD is the result of the combined action of multiple factors such as genetic susceptibility, intestinal flora imbalance, immune disorder, and environmental factors (8, 9). In the past 10 years, more than 200 gene loci associated with IBD have been identified in genome-wide association studies (GWAS) (8, 10, 11). These susceptibility gene loci are related to the barrier function, epithelial repair, microbial defence, innate immune regulation, autophagy, adaptive immune regulation, endoplasmic reticulum stress, and others (12), and are functions known to play important roles in the pathogenesis of IBD. For example, single nucleotide polymorphisms (SNPs) of the autophagy-related gene 16 like 1 (ATG16L1) are strongly associated with the risk of developing CD (13). ATG16L1 mutations results in dysfunctional autophagy, as evidenced by impaired ability of macrophages to clear intracellular bacteria and Paneth cells to secrete antimicrobial peptides (14). Furthermore, genetic variants in interleukin-10 (IL-10), IL-10 receptor(IL-10R), X-linked inhibitor of apoptosis protein (XIAP), and forkhead box P3 (FOXP3) have been linked to very early-onset inflammatory bowel disease (VEOIBD) (15). Deep sequencing studies and studies including large samples of patients with IBD are likely to generate additional knowledge on genetic variations in IBD, which will further highlight the complex genetic polymorphisms involved in this condition. Research into the gut microbiota is another area sequencing technology has been beneficial to. The general consensus is that the gut microbiota is the target of inappropriate immune response in genetically susceptible individuals, which is considered to be one of the main factors associated with the pathogenesis of IBD (16). Current evidence suggests that IBD patients have decreased diversity, altered abundance of specific taxa and functional alterations of gut microbiota (17, 18). In addition, environmental exposures due to urbanization, such as westernization of diets, increased use of antibiotics, smoking, microbial exposure and pollution can also promote intestinal inflammation in genetically susceptible individuals by affecting the intestinal microbiome, leading to the development of IBD (19, 20).

### 1.2 Immune Dysregulation in IBD

Genetic susceptibility, gut microbiota and environmental factors can contribute directly or indirectly to dysregulation of intestinal immunity. Immune dysregulation has been the focus of research into the pathogenesis of IBD because it is an autoimmune disease. The main function of the intestinal immune system, which includes innate and adaptive immunity, is to prevent the invasion of harmful pathogens while maintaining tolerance to food antigens and commensal microbes (21).

Intestinal innate immunity is the first line of defense against pathogenic invasion and consists of the intestinal epithelial barrier, innate immune cells (e.g., dendritic cells (DCs), macrophages, etc.) with their secreted cytokines and chemokines. The intestinal epithelial cells (IECs) are the main component of the intestinal epithelial barrier (IEB) and not only act as a physical barrier separating the contents of the intestinal lumen from the immune cells of the lamina propria, but also sense changes in the microenvironment of the intestinal lumen and generate local immune responses (22). Under intestinal homeostasis, the IEB remains intact and only a few luminal antigens are able to reach the lamina propria. Existing tolerance mechanisms prevent immune cells within the lamina propria from producing a pro-inflammatory immune response. However, under intestinal inflammation, increased apoptosis of IECs and reduced expression of tight junction proteins (e.g., occludin, claudins, etc.) lead to increased permeability of the IEB and further activation of intestinal innate immune cells as more luminal antigens cross the barrier, driving disease progression (23).

As the most important specialized antigen-presenting cells (APC), the main function of dendritic cells is to take up and process antigens and present antigens to T cells or B cells to regulate adaptive immune response (24). Thus, DCs play a bridging role between innate and adaptive immunity. Under intestinal homeostasis, DCs are in an immune tolerant state. However, this tolerate state is disrupted by persistent intestinal inflammation in IBD, which leads to the acquisition of a pro-inflammatory phenotype, ultimately contributing to the development of disease. In IBD patients and dextran sulfate sodium (DSS)-induced colitis mice, a large number of activated mature DCs were clustered at the inflammatory intestinal mucosa (25). These activated DCs produced high level of pro-inflammatory factors such as tumor necrosis factor alpha (TNF-α) and reactive oxygen species (ROS), which damage IECs and aggravate intestinal inflammation. Among them, TNF-α is a critical pro-inflammatory cytokine in the pathogenesis of IBD, which is up-regulated in the intestinal tissues of IBD patients, and anti-TNF-α therapy has achieved good efficacy in the treatment of IBD (23). Typically, DCs sense microbial invasion signals through the expression of pattern recognition receptors (PRRs) (e.g., toll-like receptors (TLRs), nucleotide-binding domain leucine-rich repeat receptors (NLRs), etc.) and regulate T cells-mediated adaptive immune responses through the expression of costimulatory molecules (e.g., CD40, CD80, CD86, etc.) (26). However, DCs in the intestinal tissues of IBD patients expressed significantly higher levels of TLR2 and TLR4, suggesting a stronger ability to recognize microbial antigens and contribute to disease progression (27). In addition, CD40 and CD80 expression was upregulated in DCs from IBD patients (27), which exacerbated intestinal inflammation by interacting with CD40L from T cells to produce more TNF-α, IL-6, and IL23.

Macrophages, another subtype of APC in the intestinal tissues, are usually divided into two types, classically activated (M1) and alternatively activated (M2), exhibiting two phenotypes, pro-inflammatory and anti-inflammatory, respectively (28). M1 macrophages are activated by γ-interferon (IFN-γ), TNF-α and granulocyte-macrophage colony-stimulating factor (GM-CSF), and secrete pro-inflammatory cytokines (e.g., TNF-α, IL-1β, etc.) and Chemokines (e.g., C-X-C motif chemokine ligand 9 (CXCL9), CXCL10, etc.), high expression of iNOS which could catabolize L-arginine to produce NO and ROS, participate in phagocytosis of bacteria and necrotic cells, chemotaxis of inflammatory cells, promote T helper 1 (Th1) and Th17 cells-mediated immune response. M2 macrophages are activated by IL-4, IL-13 and macrophage colony-stimulating factor (M-CSF), and not only secrete the immunosuppressive factor IL-10 to promote intestinal mucosal healing, but also recruit Tregs to inhibit intestinal inflammation. Under intestinal homeostasis, M1/M2 macrophages are in the dynamic equilibrium. However, the number of M1 macrophages was significantly increased in the intestinal tissues of patients with active IBD and mice with experimental colitis, and these increased macrophages differentiated from peripheral blood mononuclear cells (PBMCs) (29). Further, M2 macrophages have been shown to facilitate the regression of colitis by promoting angiogenesis, removing dead cells and supporting tissue repair (30). Therefore, controlling macrophage M1/M2 polarization is a potential target for the development of new therapeutic approaches for IBD.

Adaptive immune cells such as Th1-, Th2-, Th17 cells and regulatory T cells (Tregs) also play an important role in intestinal immune homeostasis. Th1 cells participate in cell-mediated immune response and are necessary for the clearance of intracellular pathogens, and mainly secrete IFN-γ, TNF-α and IL-2 (31). Th2 cells participate in humoral immune response and parasite defense, and mainly secrete IL-4, IL-5 and IL-13 (31). Treg cells can induce immune tolerance *via* secreting transforming growth factor beta (TGF-β) and IL-10 (32). Th17 cells are mainly involved in autoimmune diseases and specifically secrete IL-17 (33). It has been found that Th1 cells in CD patients secreted higher levels of TNF-α and IFN-γ compared to UC patients, and that IFN-γ promotes the secretion of TNF-α by intestinal macrophages, thereby increasing the severity of the disease (34). In contrast, Th2 cells appear to play a greater role in UC (34). Th2 cells in the mucosal tissue of UC patients secreted more IL-5 and IL-13, which promoting apoptosis of IECs and disrupting the intestinal mucosal barrier. Therefore, CD is thought to be driven by the Th1 cells-mediated immune response, whereas UC is Th2 cells. In addition, studies have shown that pro-inflammatory cytokines (e.g., IL-12, IL-18, IL-21, IL-23) were significantly increased in the inflamed intestinal mucosa of patients with IBD, while anti-inflammatory cytokines such as TGF-β and IL-10 were remarkably reduced, as well as the absence of immunomodulatory cells such as Treg cells and Foxp3^-^IL-10^+^CD4^+^ T cells (35, 36). The IL-17 produced by Th17 cells was elevated in the mucosa and serum of IBD patients (37). IL-17 not only causes damage to the IECs, but also promotes IL-8 secretion by IECs, which in turn stimulates neutrophils and Th17 cell chemotaxis to the inflamed intestinal mucosa (38).

### 1.3 Overactivation of NF-κB Signaling Pathways in IBD

**Figure 1:**
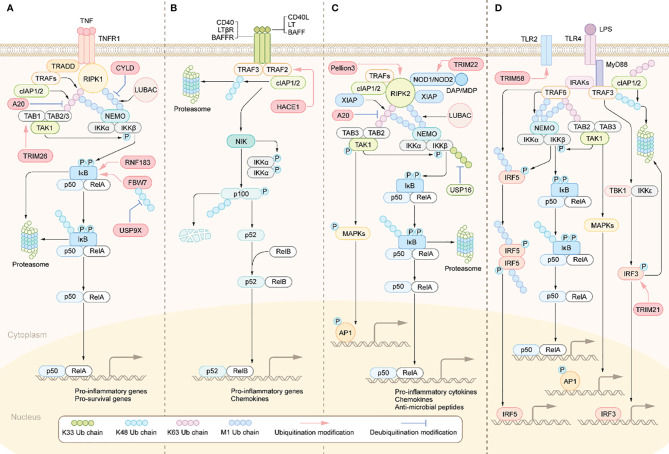
Activation and regulation of NF-κB pathway. **(A)** Canonical NF-κB activation: The binding of TNFRs to the ligands leads to the recruitment of TRADD, RIPK1. TRADD subsequently recruits E3 ubiquitin ligases, including TRAFs, cIAP1/2 and LUBAC. RIPK1 is then ubiquitinated and acts as a ubiquitin scaffold to recruit the IKK complex and the TAK1 complex. The IKK complex is comprised of IKKα, IKKβ, and NEMO. The TAK1 complex consists of TABs and TAK1.TAK1 further phosphorylates and activates IKKβ, which in turn induces phosphorylation and degradation of IκBα, allowing NF-κB dimers to translocate to the nucleus and drive transcription of target genes. **(B)** Non-canonical NF-κB activation: The interaction of the receptor with the corresponding ligand recruits TRAFs and cIAP1/2 proteins to the receptors, resulting in their ceasing to mediate the ubiquitinated proteasomal degradation of NIK, leading to stabilization and accumulation of NIK and ultimately activation of the NF-κB pathway and transcription of target genes. **(C)** NODs-mediated NF-κB and MAPKs activation pathways. DAP, MDP binds to NOD1 and NOD2 receptors in the cytoplasm, respectively, which recruit RIPK2, cIAP1/2, and XIAP. rIPK2 undergoes IAP-mediated ubiquitination modifications and acts as a ubiquitin scaffold to recruit the TAK1 complex, the IKK complex and LUBAC. subsequently, TAK1 further activates the IKKβ/IκB/NF-κB signaling cascade. In addition, TAK1 also leads to the activation of MAPKs, which induce transcription of AP1 target genes. **(D)** Activation of TLRs triggers the MYD88-dependent signaling cascade that induces NF-κB and MAPK signaling activation. MYD88 recruits IRAKs, which then activates TRAF6. TRAF6 then recruits the TAK1 complex and the IKK complex. IKKβ is then phosphorylated by TAK1, which in turn phosphorylates IκB to activate NF-κB. TAK1 also activates the MAPK signaling cascade. The signaling cascade induced by TLRs also activates IRF3 and IRF5. Activated transcription factors translocate to the nucleus and induce the production of pro-inflammatory cytokines and type I IFN.

The increased secretion of the aforementioned pro-inflammatory cytokines results in the overactivation of inflammation-related signaling pathways in IBD patients, including the nuclear factor-kappa B (NF-κB) signaling pathway (39). NF-κB activation is regulated by two different pathways, known as the canonical pathway and the noncanonical pathway (40). Inflammatory stimuli such as TNF-α and IL-1β can activate the canonical NF-κB signaling pathway (**Figure 1**). Specifically, when TNF-α binds to TNF receptors (TNFR), TNFR recruits TNFR-associated death domain protein (TRADD) to form the TNFR-TRADD complex (41). TRADD then further recruits downstream signaling proteins to form the receptor signaling complex, including receptor interacting serine/threonine kinase 1(PIPK1), TNF receptor associated factor proteins (TRAFs), cellular inhibitors of apoptosis proteins 1 and 2 (cIAP1/2) and LUBAC (linear ubiquitin chain assembly complex) (42). Subsequently, TRAFs, cIAP1/2 mediates K63-linked ubiquitination of RIPK1 and LUBAC mediates M1-linked ubiquitination of RIPK1. The K63, M1-linked ubiquitin chains on RIPK1 then acts as a scaffold to recruit the TGF-β-activated kinase 1 (TAK1) complex and the inhibitor of nuclear factor kappa B kinase (IKK) complex, respectively (43). Therefore, ubiquitination modifications on RIPK1 are a crucial step for activating this pathway. Whereafter, TAK1 phosphorylation activates IKKβ in IKK complex, which in turn phosphorylates IκB. The phosphorylated IκB is degraded though K48-linked ubiquitination modification targeting the proteasome, thus dissociating with p50/RelA, which then nuclear translocates and promotes transcription of pro-inflammatory cytokines such as TNF-α, IL-1β, and IL-6 (44). These pro-inflammatory cytokines, on the one hand, activate the adaptive immune system. On the other hand, they damage IECs and destroy the integrity of IEB. Disruption of the IEB may contribute to increased exposure of the intestinal mucosa to luminal antigens, further activating the innate and adaptive immune system, thereby perpetuating the intestinal inflammation and eventually developing chronic inflammation.

Besides TNF-α, PRRs signaling cascades can also activate NF-κB (**Figure 1**). TLRs and NLRs are members of PRR family and widely expressed in multiple cell types such as DCs, macrophages, T lymphocytes and IECs. By sensing and responding to conserved microbial-associated molecular patterns, TLRs and NLRs play a pivotal role in defence against pathogens invasion, such as bacteria, fungi and virus (45). TLRs-mediated signaling pathways can be divided into myeloid differentiation primary response protein 88 (MyD88)-dependent and MyD88-independent, of which only TLR4 is able to activate both. Specifically, following ligand-induced receptor dimerization, TLR4 interacts with MyD88 *via* TIR domain containing adaptor protein (TIRAP) to initiate the MyD88-dependent pathway (46). MyD88 further recruits IL receptor-1 receptor-associated kinases(IRAKs)and TRAF6 to form the receptor signaling complex (46). TRAF6 is then modified by ubiquitination to recruit the TAK1 complex and the IKK complex. This is followed by activation of IKKβ and mitogen-activated protein kinase (MAPK) by TAK1, and ultimately activation of the transcription factors NF-κB and AP-1, which promote transcription of various pro-inflammatory cytokines and chemokines (46). As for the MyD88-independent signaling pathway, in short, TLR4 interacts with and activates TIR-domain containing adaptor inducing interferon-β (TRIF) *via* the adaptor protein TRAM, which activating interferon regulatory factors (IRFs) and ultimately promotes transcription of interferon-related genes (47). Besides the dysregulated expression of TLRs in IBD patients mentioned above (48), the polymorphisms/mutations in some TLRs have been associated with IBD (49). Similarly, NLR family members NOD1 and NOD2, upon stimulation by different components of bacterial peptidoglycan lead to NF-κB activation through recruitment of downstream signaling proteins, including RIPK2, cIAP1/2, XIAP, TRAFs, etc. (50). Importantly, NOD2 polymorphisms/mutations are a key pathogenic event in Crohn’s disease, as NOD2 deficiency leads to exacerbated gut inflammation due to impaired bacterial clearance (51).

The noncanonical NF-κB signaling pathway can be initiated by the interaction of CD40, lymphotoxin beta receptor (LT-βR) and B-cell activating factor receptor (BAFFR) with their corresponding ligands (**Figure 1**). Under these stimuli, the complex composed of TRAF2/3 and cIAP1/2 will be recruited to the intracellular side of the receptor. Therefore, TRAF2/3 and cIAP1/2 no longer mediate the ubiquitin degradation of NF-κB inducing kinase (NIK) and thus activates NIK (52). Then NIK results in activation of IKKα, phosphorylation and proteasomal degradation of p100, activation of the transcription factor NF-κB (p52/RelB) and ultimately promotes transcription of pro-inflammatory cytokines and chemokines (53). Studies have found that p100/p52 was markedly upregulated in the intestinal tissues of IBD patients, suggesting increased activation of the non-canonical NF-κB signaling cascade (54).

### 1.4 Animal Models of IBD

To better elucidate the interplay among the pathogenic factors described above, animal models of IBD are essential. To date, more than 100 animal models of IBD have been established, which can be divided into chemical induced models, genetically engineered models, adaptive cell transferred models and congenital (spontaneous gene mutation) models (55). Chemical induced IBD animal models are one of the most widely used types at present, among which DSS and TNBS are the most common. DSS has been widely used in acute and chronic colitis models, and is well suited to study the initial stages of inflammation and the healing process of intestinal epithelium during recovery stage (56). Of note, based on DSS-induced chronic colitis model, inflammation-related colorectal cancer can be caused by controlling the duration and number of repetitions of DSS administration (e.g., 7 days DSS, 14 days water, 3 cycles) and/or in combination with genotoxic colonic carcinogen azoxy methane (AOM) (57). Since chronic inflammation plays a key role in colitis-associated colorectal cancer, the AOM/DSS model is a very useful tool for studying chronic inflammation-induced carcinogenesis. The 2,4,6-trinitrobenzenesulfonic acid (TNBS)-induced colitis resembles human Crohn’s disease, which has the advantages of short modelling time and long lesion duration, so it is suitable for observing the dynamic process of inflammation from acute phase to chronic stage (58). The disadvantage is that compared with DSS-induced model, TNBS-induced colitis lacks obvious acute phase and is highly dependent on mouse strains. For example, SJL/J, C3HeJ and BALB/c are sensitive strains, while C57BL/6 and DBA/2 are highly resistant strains (59). In recent years, genetically engineered IBD animal models have developed rapidly, and there are many classifications, among which gene knockout models (including conventional knockout and cell-specific knockout) and transgenic models (including conventional and cell-specific genotyping) are the most used (60). Simply put, conventional transgenic (Tg) or knockout (KO) mice were genetically engineered to overexpress or lack genes of interest in all cell types. Cell-specific Tg or KO models overexpressed or lacked genes of interest in specific cell types, respectively (60). Compared with chemical induced models, genetically engineered animal models have unique advantages: they can be modified (deletion or overexpression) for one or several specific genes, and clearly clarify the role of these genes in the occurrence and development of IBD. However, genetically engineered animal models also have some disadvantages: high technical content and production costs, knock out some of the essential genes may cause the lethality of the cells or animals, and knock out a gene does not necessarily can learn the function of the gene, mainly because many of the genes are functionally redundant. Knocking out a functionally redundant gene does not create an easily identifiable phenotype. This is why more and more genetically engineered models and chemical induced models are combined used. The adaptive cell transferred model (e.g., CD4^+^CD45RB^high^ T cells) is one of the models to mimic chronic colitis in which naive T cells from immunoactivity mice are transferred to T - and B-deficient hosts (e.g., *Rag1/2 ^-/-^* or SCID mice) to induce colonic inflammation (61). This model is suitable for observing how different types of T cells participate in the occurrence and development of IBD, but it requires flow cytometry purification and certain intravenous injection skills (61). Some animals in nature can spontaneously develop enteritis similar to human IBD, and such enteritis model is regarded as congenital (spontaneous gene mutation) models, among which *Mdr1a^-/-^* mice, C3H/HEJBIR(C3BIR) mice, SAMP1/YitFc mice are more common (62). *Mdr1a^-/-^* mice are considered to be a more accurate model for studying human UC, while the SAMP1/YitFc mice model can be used as a closed CD model, showing perianal disease and fistula formation in about 5% of mice (63, 64). In general, there is no single animal model that can fully mimic the onset of human IBD, although there are many options but each has its own advantages and disadvantages. Therefore, the establishment of animal models more closely related to human IBD is of great significance to elucidate the pathogenesis of IBD and promote clinical diagnosis and treatment as well as the development of new drugs.

## 2 Ubiquitination and Deubiquitination in IBD

**Figure 2:**
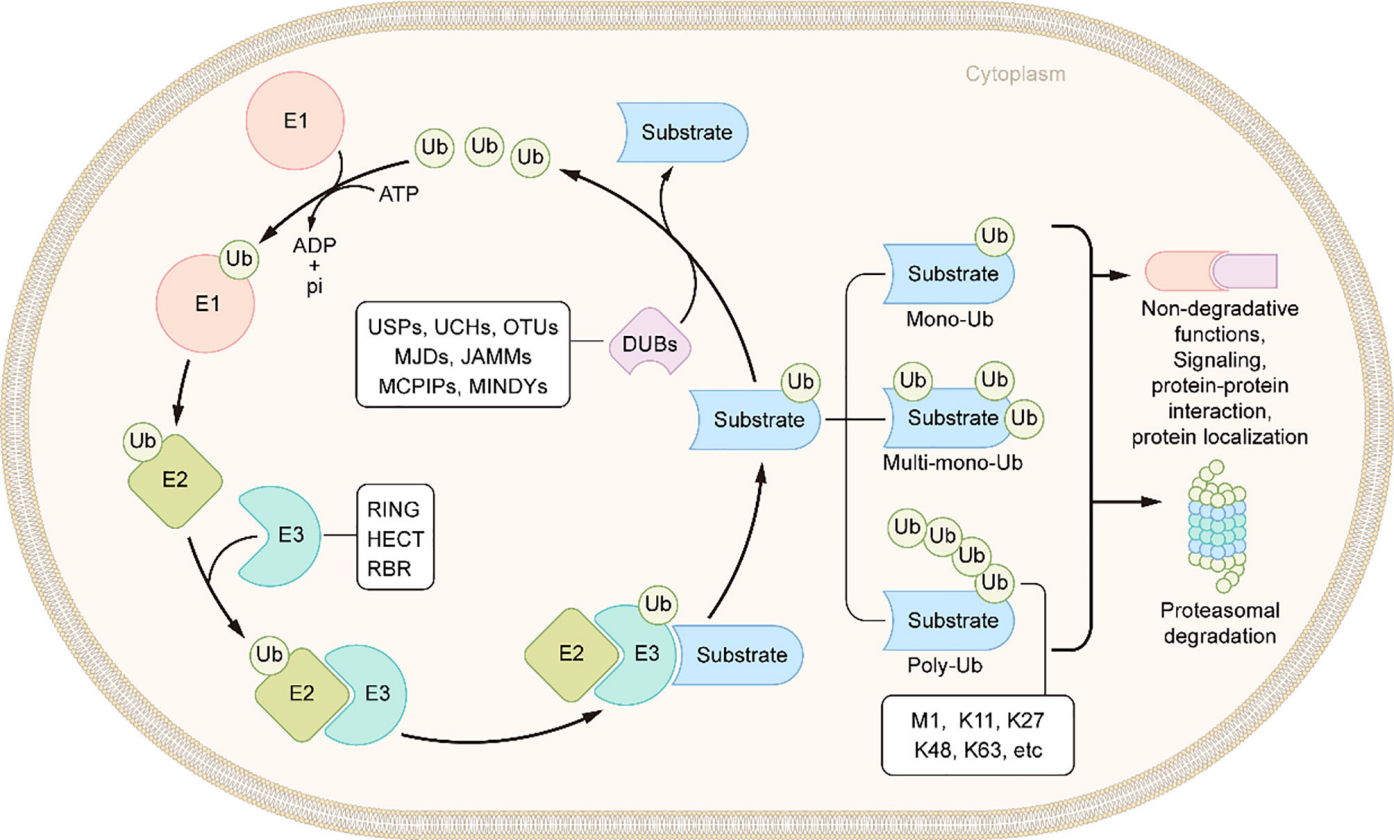
Schematic of ubiquitination and deubiquitination. In an ATP-dependent way, the E1 enzyme activates ubiquitin, forming a covalent thioester connection between ubiquitin and the E1 cysteine residue. Then, ubiquitin is transported to an E2 conjugating enzyme. Finally, an E3 ligase aids or catalyzes the transfer of ubiquitin from an E2 to a substrate, generally through a lysine side chain. DUBs remove the ubiquitin molecules from the substrates.

**Figure 3:**
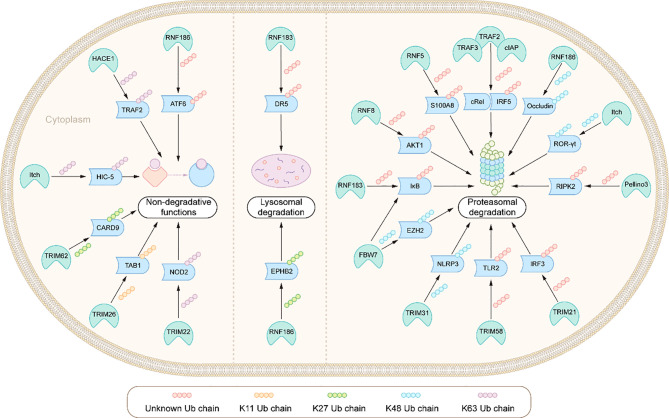
Integration: E3s ligases and their ubiquitination modified substrates in the pathogenesis of IBD.

Ubiquitin is a small protein of 76 amino acid residues. Ubiquitin, as its name suggests, is widely distributed in eukaryotic cells and tissues. Ubiquitin modification involves an ATP-dependent enzymatic cascade of ubiquitin molecules covalently linked to substrate proteins, and is mediated by three types of enzymes: ubiquitin activating enzymes (E1s), ubiquitin binding enzymes (E2s), and ubiquitin ligases (E3s) (65). In short, E1 first hydrolyzes a molecule of ATP and activates a ubiquitin molecule, then the activated ubiquitin molecule is transferred to E2, and, finally, E3 promotes or directly catalyzes ubiquitin transfer to the lysine residues of the substrate protein by recruiting the E2-ubiquitin complex which recognizes the substrate protein (**Figure 2**) (66). Ubiquitination is divided into mono-ubiquitin and polyubiquitin (ubiquitin chain) according to the number of ubiquitin molecules linked to a lysine residue in a protein. In the polyubiquitin chain, ubiquitin can be linked by seven lysine residues (K6, K11, K27, K29, K33, K48 and K63) or by the first methionine (M1) (67). The results of the ubiquitin modified protein depend on the type of ubiquitin chain link. Generally, K48 and K11 linked polyubiquitin chains represent the target signals for proteasomal degradation, while K63 linked polyubiquitin chains are associated with non-proteasomal signals, including cell signal transduction, DNA damage response, and membrane transport (68).The human genome encodes 2 E1s, more than 50 E2s and 600 E3s (69, 70). E3 ubiquitin ligases, which play a key role in the whole process of ubiquitination due to its substrate specificity, can be divided into three types: really interesting new gene (RING) E3s, homologous to E6AP carboxyl terminus (HECT) E3s, and ring-in-between-ring (RBR) E3s (71, 72). These three types of E3s mediate ubiquitination *via* different mechanisms: RING E3s transfer ubiquitin directly from E2s to the substrate protein using its ring-finger domain (73), HECT E3s receive ubiquitin from E2s to form catalytic intermediates before transferring ubiquitin to the substrate protein (74, 75), and RBR E3s use both RING and HECT like mechanisms (76). RING E3s and HECT E3s have been relatively well studied in the context of IBD and will be described in detail below (**Table 1** and **Figure 3**). However, studies on RBR E3s in the context of IBD are rare and the function of these enzymes will need to be further clarified in future studies.

**Table 1:** Role of E3 ubiquitin ligases in inflammatory bowel disease.

Gene	Effect	Relevance to IECs or immune cell components	Alteration in IBD patients	Transgenic mice model	Experimental colitis model	Disease Phenotype	Reference
TRAF2	Anti-inflammatory	IECs, IL-10-secreting neutrophils, macrophages	Up-regulated in intestinal mucosa	*Traf2^-/-^*, *Traf2^Myeol-KO^*	DSS	Spontaneous colitis; Severe colitis	(77–80)
TRAF3	Anti-inflammatory	PBMCs, IECs	Up-regulated in PBMC and colonic mucosa	*Traf3^Myeol-KO^*	DSS	Severe colitis	(79, 81, 82)
TRAF4	Unknown	PBMCs, IECs	Up-regulated in plasma, PBMC and intestinal mucosa	-	-	-	(83)
TRAF5	Anti-inflammatory	PBMCs, CD4^+^T	Up-regulated in PBMC and colonic mucosa	*Traf5^-/-^*	DSS	Susceptibility to colitis	(81, 84)
TRAF6	Anti-inflammatory	IECs	Up-regulated in plasma, PBMC and intestinal mucosa	*Traf6^IEC-KO^*	DSS	Susceptibility to colitis	(83, 85)
TRIM14	Pro-inflammatory	THP-1, BMDM, PBMCs	-	*Trim14^-/-^*	DSS, DSS/AOM	Attenuated colitis, inhibit colitis-associated tumorigenesis	(86)
TRIM21	Anti-inflammatory, inhibit colitis-associated tumorigenesis	CD4^+^T cells	Down-regulated in intestinal mucosa of IBD, CAC and CRC	*Trim21^-/-^*	DSS, DSS/AOM, CD45RB^high^ cells	Susceptibility to colitis and CAC	(87, 88)
TRIM26	Anti-inflammatory	Macrophages	-	*Trim26^-/-^*	DSS	Attenuated colitis	(89)
TRIM27	Pro-inflammatory, promote colitis-associated tumorigenesis	CD4^+^T cells	Down-regulated in colonic mucosa of CD	*Trim27^-/-^*		Attenuated colitis, inhibit colitis-associated tumorigenesis	(90–93)
TRIM31	Anti-inflammatory	Macrophages	-	*Trim31* ^-/-^	DSS	Attenuated colitis	(94)
TRIM33	Anti-inflammatory	PBMCs, myeloid cells	Down-regulated in PBMC of CD	*Trim33^-/-^*	DSS	Severe colitis	(95)
TRIM34	Anti-inflammatory, inhibit colitis-associated tumorigenesis	IECs	Down-regulated in colonic mucosa of UC	*Trim34^-/-^*	DSS, DSS/AOM	Attenuated colitis, inhibit colitis-associated tumorigenesis	(96)
TRIM58	Anti-inflammatory	Myeloid cells	Down-regulated in colonic mucosa of UC	*Trim58^-/-^*	DSS	Susceptibility to colitis	(97)
TRIM62	Anti-inflammatory	Dendritic cells	-	*Trim62^-/-^*	DSS	Severe colitis	(98)
RNF5	Anti-inflammatory	IECs, CD4^+^T cells	Down-regulated in intestinal mucosa	*Rnf5^-/-^*	DSS	Severe colitis	(99)
RNF8	Anti-inflammatory	IECs	-	LV-RNF8 (overexpressing RNF8)	TNBS	Attenuated colitis	(100)
RNF20	Anti-inflammatory, inhibit colitis-associated tumorigenesis	IECs, monocytes and macrophages, MDSCs	Down-regulated in colonic mucosa of UC and CAC	*Rnf20^+/-^*	DSS, DSS/AOM	Susceptibility to colitis and CAC	(101)
RNF40	Pro-inflammatory	IECs	Down-regulated in colonic mucosa of UC and CAC	*Rnf40^IEC-KO^*	DSS	Attenuated colitis	(101, 102)
RNF128	Unknown	CD4^+^T cells	Up-regulated (lamina propria CD4^+^T), down-regulated (PB CD4^+^T)	-	-	-	(103, 104)
RNF183	Pro-inflammatory	IECs	Up-regulated in intestinal mucosa	-	-	-	(105, 106)
RNF186	Anti-inflammatory	IECs, macrophages,	Down-regulated in colonic mucosa of UC	*Rnf186^-/-^*	DSS	Susceptibility to colitis	(107, 108)
cIAP1	Unknown	IECs, macrophages,	-	*Bric2^-/-^*	-	Susceptibility to TNF-induced cell death	(109–111)
cIAP2	Anti-inflammatory, promote colitis-associated tumorigenesis	IECs, macrophages	Up-regulated in colonic mucosa of UC	*Bric3^-/-^*	DSS, DSS/AOM	Susceptibility to colitis, inhibit colitis-associated tumorigenesis	(109–114)
XIAP	Unknown	IECs, macrophages	-	*Bric4^-/-^*	-	-	(111, 115)
FBW7	Anti-inflammatory^IEC^, pro-inflammatory^Myeol^	IECs, macrophages	Up-regulated in intestinal mucosa	*Fbw7^IEC-KO^*, *Fbw7^LysM+-KO^*	DSS, TNBS	Severe colitis, Attenuated colitis	(116–118)
PELLINO3	Anti-inflammatory	Macrophages	Down-regulated in colonic mucosa of CD	-	-	-	(119)
HRD1	Anti-inflammatory	Unknown	Down-regulated in intestinal mucosa	-	-	-	(120, 121)
ITCH	Anti-inflammatory	Th17 cells, ILCs, γδT cells	-	*Itch^-/-^*	DSS, DSS/AOM	Spontaneous colitis, Susceptibility to colitis and CAC	(122–125)
HACE1	Anti-inflammatory	IECs	-	*Hace1^-/-^*	DSS, DSS/AOM	Susceptibility to colitis and CAC	(126)

IBD, inflammatory bowel disease; CD, Crohn’s disease; UC, ulcerative colitis; CAC, colitis-associated colorectal cancer; CRC, colorectal cancer; PB, peripheral blood; PBMC, peripheral blood mononuclear cell; MDSC, myeloid-derived suppressor cells; BMDM, bone marrow-derived macrophages; ILCs, Innate lymphoid cells; DSS, dextran sulfate sodium salt; TNBS, trinitrobenzene sulfonic acid; AOM, azoxymethane.

**Figure 4:**
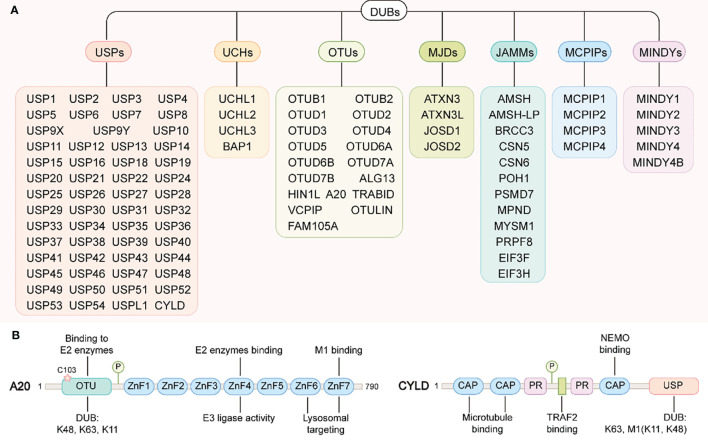
DUBs family and the structural domains of A20 and CYLD. **(A)** DUBs family. DUBs can be divided into seven types: ubiquitin c-terminal hydrolases (UCHs), ubiquitin-specific proteases (USPs), ovarian tumor-related proteases (OTUs), Machado-Joseph disease protein domain proteases (MJDs), Jab1/MPN domain-associated metalloisopeptidase(JAMMs), monocyte chemotactic protein-induced proteins (MCPIPs) and motif interacting with ub-containing novel DUB family(MINDYs). **(B)** A20 structural domains(left): A20 contains an N-terminal OTU domain responsible for the DUB activity of A20. The catalytic cysteine residue Cys103 is also important for binding to the E2 enzymes. A20 contains seven zinc finger domains in its C-terminus. ZnF4 confers A20 E3 ligase activity, where as ZnF7 have been essential for A20 binding to M1-linked ubiquitin chain. In addition, ZnF6 and ZnF7 are required for A20 targeting to lysosomes. CYLD structural domains(right): the N-terminal of CYLD contains three CAP structural domains and two proline-rich (PR) motifs, of which the first two CAP domains are responsible for binding to microtubules and the third interacts with NEMO (also called IKKγ). Between the two PR motifs there is one binding site to TRAF2. The C-terminus of CYLD contains a USP structural domain responsible for its DUB activity.

Like most post-translational modifications, ubiquitin modification is also a dynamic and reversible process. This process is catalyzed by deubiquitinases (DUBs), which specifically remove ubiquitin molecules from substrate or precursor proteins by hydrolyzing ester bonds, peptide bonds or isopeptide bonds at the carboxyl terminal, to avoid degradation or reverse other functional changes caused by ubiquitination(**Figure 2**) (127). There are more than 100 DUBs encoded by the human genome, and these can be divided into seven types according to their similarity in sequence and structure: ubiquitin c-terminal hydrolases (UCHs), ubiquitin-specific proteases (USPs), ovarian tumor-related proteases (OTUs), Machado-Joseph disease protein domain proteases (MJDs), Jab1/MPN domain-associated metalloisopeptidase(JAMMs), monocyte chemotactic protein-induced proteins (MCPIPs) and motif interacting with ub-containing novel DUB family(MINDYs) (**Figure 4**) (128, 129). Except for JAMMs, which are zinc-dependent metalloproteinases, the other DUBs are cysteine-dependent proteases (130). Mainly through deubiquitination of its substrate proteins, DUBs are involved in the regulation of various cellular activities, including cell cycle, signal transduction, DNA damage repair, gene transcription, autophagy and apoptosis (131–133). In conditions of intestinal inflammation, many DUBs promote or inhibit inflammation by controlling protein stability, the formation of intermediate signal molecules or by affecting receptor activity. Therefore, DUBs might have potential as drug targets, and hold broad clinical application prospects. In this review, we review the literature on USPs and OTUs family members in the context of IBD (**Table 2**).

**Table 2:** Role of DUBs in inflammatory bowel disease.

Gene	Effect	Relevance to IECs or immune cell components	Alteration in IBD patients	Transgenic mice model	Experimental colitis model	Disease Phenotype	Reference
CYLD	Anti-inflammatory	B cells, T cells, myeloid cells, IECs	Down-regulated in intestinal mucosa	*Cyld^-/-^*, *IEC-Cyld* ^Δ9^, *scyld/Smad7*	DSS, DSS/AOM C.rodentium	Susceptibility to colitis and CAC, severe colitis	(134–138)
USP8	Anti-inflammatory	T cells	-	*Usp8^fl/fl^ CD4-Cre*	-	Spontaneous colitis	(139)
USP9X	Anti-inflammatory, inhibit colitis-associated tumorigenesis	IECs	Down-regulated in intestinal mucosa of CRC	*Usp9x^fl/fl^ Villin-Cre*	DSS, DSS/AOM	Severe colitis, susceptibility to CAC	(140)
USP16	Pro-inflammatory	Macrophages	Up-regulated in colonic macrophages	*Usp16^fl/fl^ Lyz2-Cre^+^*	DSS, DSS/AOM	Attenuated colitis, inhibit colitis-associated tumorigenesis	(141)
USP22	Anti-inflammatory, inhibit colitis-associated tumorigenesis	IECs	Down-regulated in colonic mucosa of UC and UC^neo^	*Usp22^fl/fl^ Villin-CreER^T2^*	DSS	Severe colitis, susceptibility to CAC	(142, 143)
USP38	Anti-inflammatory	BMDCs	-	*Usp38^-/-^*	DSS	Susceptibility to colitis	(144)
A20	Anti-inflammatory	IECs, dendritic cells, myeloid cells	Down-regulated in intestinal mucosa (protein level)	*A20^-/-^*, *A20 Tg*, *A20^IEC-KO^*, *A20^fl/fl-^Myd88* ^fl/fl^, *A20^fl/fl^ Cd11c-Cre, A20^fl/fl^ Cd11c-Cre Rag1, A20^Myel-KO^, A20^OTU^, A20^ZF4^, A20^ZnF4ZnF7/ZnF4ZnF7^, A20^ZnF4ZnF7/ZnF4ZnF7^Vil1-Cre, A20^ZnF4ZnF7/ZnF4ZnF7^LysM-Cre*	DSS	Spontaneous colitis, susceptibility to colitis	(145–159)
OTUD5	Pro-inflammatory	LPMCs	Up-regulated in intestinal mucosa	-	-	-	(160)

DUBs: deubiquitinases; UC: ulcerative colitis; CAC: colitis-associated colorectal cancer; CRC: colorectal cancer; DSS: dextran sulfate sodium salt; AOM: azoxymethane; BMDCs: bone marrow-derived dendritic cells; LPMCs: lamina propria mononuclear cells.

## 3 Ub E3 Ligases and IBD

### 3.1 The Role of RING-Type E3s

#### 3.1.1 TRAFs

The tumour necrosis factor receptor-associated factor (TRAF) family is composed of cytoplasmic adaptor proteins involved in the transduction of downstream signals of various receptors, such as T cell receptors (TCRs), TLRs, NLRs, and IL-17 receptor (IL-17R) (161). To date, seven members of the TRAFs family have been identified, which, except for TRAF1, contain in the N-terminus a ring finger domain, the domain responsible for the catalytic activity of E3 ubiquitin ligase (162). Recent studies have also demonstrated that TRAF2, TRAF3, TRAF5 and TRAF6 function as E3 ubiquitin ligases (161). TRAFs, as adaptor proteins and E3 ubiquitin ligases, are involved in innate and adaptive immune signal transduction, leading to the activation of transcription factors such as NF-κB, AP-1and IRFs (163).

Preliminary evidence linking TRAFs with intestinal inflammation was suggested by a study reporting that *Traf2* ^-/-^ mice, with BALB/C background, spontaneously developed severe colitis and died within 3 weeks after birth (77). The spontaneous colitis phenotype was largely dependent on TNF-α induced apoptosis of colonic epithelial cells. The production of TNF-α by colon lamina propria cells (LPCs) in response to symbiotic bacteria may have been a key event that exacerbated the development of colitis, due to increasing apoptosis of colonic epithelial cells and aggravation of the destruction of epithelial barrier function, leading to dramatic alterations in colonic microecology (77). Changes in colonic microbiota will also cause LPCs to further produce TNF-α, which will then lead to uncontrolled intestinal inflammation (77). Subsequently, *Traf2^-/-^* mice were also found to have abnormal accumulation of IL-10-secreting neutrophils, a finding that was not surprising given that development of IL-10-secreting neutrophils is largely dependent on TNF-α signaling (78). IL-10-secreting neutrophils might induce immunosuppression under certain conditions and aggravate colitis by enhancing colonic bacterial invasion, suggesting a potentially important role for TRAF2-mediated TNF-α signaling in regulating IL-10-mediated colonic homeostasis. Additional studies found that myeloid cell-specific knockout of TRAF2 or TRAF3 aggravated colitis by promoting expression of pro-inflammatory cytokines stimulated by TLRs in macrophages (79). Investigation of the molecular mechanisms suggested that TRAF2 and TRAF3 acted synergically with E3 ubiquitin-ligase cAIPs to mediate ubiquitination of cRel and IRF5. Following TRAF2 and TRAF3 knock-out, cRel and IRF5 achieved stable expression levels, rendering macrophages highly sensitive to TLR ligands and IL-1β-induced cytokines (79). These results indicate an involvement of TRAF2 and TRAF3 in the negative feedback regulation mechanism of inflammation suppression. Another study from Jun, Qiao et al. observed TRAFs up-regulation in PBMC and colonic mucosa of IBD patients, further suggesting a role for this family of proteins in the development and progression of IBD (80, 81, 83). In addition, TRAF3 has been shown to be a negative regulator of inflammation in the TNBS-induced colitis mouse model by interfering with IL-17/IL-17R/Act1/TRAF6-mediated proinflammatory pathways though binding to IL-17R (82). Furthermore, increased sensitivity to DSS-induced colitis was observed in *Traf5^-/-^* mice, and might be attributed to enhanced Th2 and IFN-γ/IL-17A co-producing CD4^+^T cell responses and CD4^+^T cell NF-κB activation under intestinal inflammation (84). These findings suggest that TRAF5 works as an anti-inflammatory regulator in experimental colitis in mice. Similarly, specific knockout of TRAF6 in IECs also leads to severe DSS-induced colitis in mice, suggesting that TRAF6 exhibits protective anti-inflammatory effect in intestinal epithelial cells; it is worth noting that this effect appears to be independent of TLR signals (85).

Overall, TRAFs showed a consistent anti-inflammatory effect during the occurrence and development of IBD, albeit *via* different pathways or target cells. How TRAFs play an anti-inflammatory role in IBD through the regulation of innate immunity and adaptive immunity remains an issue worthy of further exploration. Another question to be explored in future studies is how to separately evaluate the different roles of each TRAF protein in the separation of other functions.

#### 3.1.2 TRIMs

Most member of the tripartite motif protein superfamily (TRIMs) exhibit E3 ubiquitin ligase activity due to the presence of ring finger domain, which is involved in the regulation of a variety of cell biological processes, including cell homeostasis, cell cycle, apoptosis, senescence (164). Increasing evidence suggests that some TRIM family members promote or inhibit the development of IBD through different mechanisms. Firstly, knockout of TRIM14 alleviated acute colitis and CAC by weakening the nonclassical NF-κB pathway mediated inflammatory response; this is due to the ability of TRIM14 to recruit USP14 to deubiquitination p100/p52, thereby preventing it from p62 mediated autophagy degradation (86). Secondly, TRIM21 was found to inhibit Th1/Th17 differentiation in the intestinal mucosa by ubiquitinating IRF3 in CD4^+^T cells, thus playing an anti-inflammatory role in the pathogenesis of IBD (87). Similarly, Zhou et al. reported that TRIM21 expression in colitis-associated colorectal cancer (CAC) decreased and was negatively associated with colon cancer occurrence, further suggesting an anti-cancer effect of TRIM21 (88). Subsequently, TRIM22 mutants have been reported to be impaired in their ability to bind NOD2 and mediate NOD2 K63-linked polyubiquitination, leading to NOD2-dependent activation of IFN-β and NF-κB signals, which were involved in the occurrence of with VEOIBD (165). In addition, TRIM26-mediated K11-linked TAB1 polyubiquitination has been found to enhance TAK1 activation and subsequent activation of NF-κB and MAPK signaling pathways in macrophages, pointing to a pro-inflammatory role of TRIM26 in DSS-induced colitis (89). Furthermore, TRIM27 expression was elevated in the colon tissues of Crohn’s patients and in CD4^+^T cells in the mesenteric lymph nodes of DSS-induced colitis mice (90, 91). In a study by Zaman et al., *Trim27^-/-^* mice were not sensitive to DSS-induced colitis, again implicating TRIM27 in the occurrence and development of IBD (92). Further research reported that the knockout of TRIM27 reduced DSS induced intestinal inflammation and inhibited tumorigenesis of CAC induced by AOM/DSS, mainly due to repression of signal transducer and activator of transcription 3 (STAT3) activation in hematopoietic cells (93). However, hyperactivation of STAT3 has been recognised as an important mechanism in the transition from colonic inflammation to colonic neoplasia (166). These results suggest that TRIM27 is not only a pro-inflammatory factor, but also a potential oncoprotein. Moreover, TRIM31 was found to suppress the activation of NLRP3 inflammasome by promoting NLRP3 polyubiquitination and proteasome degradation, thus alleviating DSS-induced colitis (94). Additionally, research by Petit et al. showed down-regulation of TRIM33 expression in PBMC of CD patients, and that specific knockout of TRIM33 in myeloid cells caused impaired monocyte recruitment and macrophage differentiation, leading to a continuous state of colonic inflammation (95). These results indicated that expression of TRIM33 in myeloid cells was important in the maintenance of intestinal immune homeostasis. More recently, the expression level of TRIM34 was also found to be significantly decreased in the colonic mucosa of patients with UC (96). TRIM34 knock-out contributed to decreased secretion of Muc2 by goblet cells, leading to defects in the internal mucus layer. This phenotype makes mice more susceptible to DSS-induced colitis and inflammation-associated colorectal cancer, suggesting that TRIM34 in IECs plays an important role in maintaining the integrity of intestinal barrier, and in preventing severe colitis and tumorigenesis (96). Furthermore, mRNA and protein levels of TRIM58 were significantly reduced in colon tissues of mild or active UC patients (97). Additional mechanistic studies revealed that TRIM58 regulates TLR2 in myeloid cells by ubiquitination, and inhibits intestinal inflammation though terminating the overactivation of NF-κB/AP-1 signaling pathway induced by TLR2 (97). Finally, *Trim62^-/-^* mice exhibited reduced cytokine production dependent on caspase recruitment domain-containing protein 9 (CARD9) signaling pathway, and increased susceptibility to fungal infection and DSS-induced colitis (98). CARD9 is a susceptibility gene for IBD and a critical component of anti-fungal innate immune signaling (167). Further research found that TRIM62 mediated K27-linked ubiquitination of CARD9 at K125 site was critical for CARD9 activation in DCs, suggesting an involvement of TRIM62 in mucosal anti-fungal immune response and intestinal inflammation (98).

In conclusion, the TRIM protein family play important roles in the development of IBD by regulating innate and adaptive immune systems. More research is needed to elucidate the exact mechanisms by which members of the TRIM family influence the occurrence or progression of inflammation at a molecular level. It will be interesting to see whether these processes occur in an E3 ubiquitin ligase activity dependent manner.

#### 3.1.3 Other Ring-Type E3 Ligases

Ring finger protein 5 (RNF5) expression was found to be decreased in the colonic inflammatory mucosa of IBD patients, and was negatively associated with S100 calcium binding protein A8 (S100A8) expression (99). S100A8 can induce neutrophils chemotaxis as well as promoting the expression of pro-inflammatory cytokines in immune cells (168). The inverse correlation of RNF5/S100A8 was consistent with the clinical severity of IBD patients, suggesting that the RNF5/S100A8 axis may play a role in the development of IBD (99). Studies using animal models found that RNF5 deletion aggravated DSS-induced colitis, increased the production of Th1-type inflammatory cytokines, and impaired intestinal epithelial regeneration in the inflammatory recovery stage (99). Further mechanistic studies revealed that lack of RNF5 in IECs weakened mediated ubiquitination and proteasomal degradation of S100A8, which in turn promoted S100A8 secretion and induced activation of CD4^+^T cells (99). Decreased expression of RNF8 in colonic tissue was associated with impaired autophagy and elevated levels of phosphorylated Akt/mTOR in the TNBS-induced colitis mouse model (100). Overexpression of RNF8 reversed these phenotypes and reduced intestinal inflammation, possibly due to RNF8 ubiquitin degradation of AKT1 and inhibition of Akt/mTOR signal pathway to enhance autophagy (100). In addition, the expression of RNF20 and RNF40 was down-regulated in the colonic epithelium and in the stroma of UC and colitis-associated colorectal cancer (CAC) patients (101). The susceptibility of RNF 20 heterozygote (*Rnf20^+/-^*) mice to colitis and inflammatory-associated tumour was increased, likely because RNF20 deletion in non-cancerous epithelial cells, intestinal organoids, and innate immune cells promoted p65 binding to the κB site, leading to the transcriptional activation of NF-κB target genes (101). Intestinal epithelial cells-specific knockout of RNF40 resulted in local and systemic protective effects on DSS induced colonic inflammation, and these effects were different from the anti-inflammatory and tumour suppressive effects of RNF20 (102). RNF40 deficiency in IECs not only reduced the burden of colonic inflammation by reducing NF-κB transcription activity *via* delaying the nuclear translocation of RelA, but also alleviated bone fragility induced by inflammation (102). RNF128 was also found to be involved in the pathogenesis of IBD. On one hand, RNF128 expression was found to be up-regulated in the CD4^+^T cells of the intestinal layer propria in CD patients, while down-regulated in CD4^+^T cells of the peripheral blood (103). On the other hand, the expression of RNF128 in CD4^+^T cells in UC patients in remission stage was significantly higher than patients with active UC and healthy subjects, indicating that RNF128 may be involved in maintaining remission in UC patients (104). Furthermore, the expression of RNF183 was up-regulated in IECs of IBD patients and TNBS-induced colitis mouse model, and promoted NF-κB signaling mediated intestinal inflammation by increasing ubiquitination -proteasome degradation of IκB (105). Similarly, RNF183 expression in colonic epithelial cells was up-regulated in IBD patients and DSS-induced colitis mouse model, leading to caspase-8 mediated apoptosis by promoting K63-linked ubiquitination-mediated lysosomal degradation of death receptor 5 (DR5) (106). Consistent with this, DR5 was downregulated in IECs of IBD patients (169). Previous research has shown that translocation of DR5 to lysosomes leads to the release of lysosomal proteases into the cytoplasm, thereby promoting apoptosis (170). These findings imply that RNF183 may play a pro-inflammatory role in the context of IBD.

Notably, GWAS identified RNF186 as a UC susceptibility gene (171). Further studies have shown that R179X, a truncated mutant of RNF186, had a protective effect on UC, possibly due to R179X mislocalization and impaired RNF186 function or altered associations with interacting proteins and subsequent substrate protein ubiquitination (172). Besides, the UC-associated variation (A64T, substitution of alanine with threonine at the 64th position) of RNF186, which was identified in North American and European UC patients (173), impaired the E3 ubiquitin ligase activity of RNF186 and was associated with increased sensitivity to DSS-induced intestinal inflammation in mice (107). Meanwhile, *Rnf186^-/-^* mice showed increased colon permeability to organic solutes and high sensitivity to DSS-induced colitis (107). The reason for increased colon permeability might be due to the role of RNF186 in controlling occludin homeostasis through the K48-linked polyubiquitination, hence RNF186 absence will result in increased amounts of occludin and abnormal distribution (concentrated in the cytoplasm) in IECs (107). With respect to high sensitivity to DSS-induced colitis, RNF186 deficiency led to disturbed proteostasis and thus increased endoplasmic reticulum (ER) stress in IECs (107). Recently, it has been reported that RNF186 knockout mice were found to have increased bacterial loads in their mesenteric lymph nodes and spleen during DSS-induced colitis, and RNF186-deficient macrophages were impaired in bacterial phagocytosis and intracellular bacterial clearance (108). Further mechanistic studies revealed that the ER localization of RNF186 in macrophages and its mediated ubiquitination of activating transcription factor 6 (ATF6) were crucial steps in NOD2-induced antimicrobial effect (108). In addition, increased sensitivity to DSS colitis in *Rnf186^-/-^* mice was linked to reduced autophagy in the colonic epithelia attributed to RNF186 mediating K27-linked ubiquitination of EphB receptor B2 (EPHB2) at K892 site and further recruiting MAP1LC3B for autophagy (174). This RNF186-dependent, EPHB2-induced autophagy helped to promote the clearance of bacteria from the colonic epithelium (174). These results suggest a clear role for RNF186 in regulating intestinal homeostasis, albeit through different regulatory mechanisms.

cIAP1, cIAP2 and XIAP, members of the inhibitor of apoptosis (IAP) family, were initially identified as anti-apoptotic proteins. However, accumulating studies have shown that cIAP1, cIAP2 and XIAP are key and universal regulatory factors in inflammatory and innate immune signaling pathways, which is attributed to their E3 ubiquitin ligase activity (43). For example, XIAP is indispensable for NOD-mediated NF-κB activation. Briefly, the research conducted by Bauler et al. showed that XIAP facilitated maximal production of pro-inflammatory cytokines during bacterial infection *in vivo* and *in vitro*, or during combined treatment with NOD2 and TLR2 ligands, taking the lead in suggesting a role for XIAP in NOD signaling (175). Subsequently, Krieg et al. revealed that XIAP mediated NOD signaling by interacting with RIPK2 *in vitro (* 176). Furthermore, Damgaard et al. demonstrated that XIAP was an essential ubiquitin ligase in the NOD2 signaling pathway *in vivo (* 115). Mechanistically, Once the NOD receptors activated by specific PAMPs, XIAP is recruited to the NOD receptor signaling complex (NOD-SC) containing RIPK2 (115). On the one hand, XIAP promotes the ubiquitination of RIPK2, which is a critical step in NOD-mediated NF-κB activation (115). On the other hand, XIAP recruits LUBAC to the NOD-SC to further activate the NOD downstream signaling cascade (115). Therefore, dysregulation of XIAP results in impaired NOD signal transduction, reduced response to bacteria and increased intestinal inflammation. Indeed, numerous studies have shown that XIAP mutations are associated with IBD (177). XIAP mutations may cause a primary immunodeficiency disease, X-linked lymphoproliferative disease type 2 (XLP-2), which is often characterized by hemophagocytic lymphohistiocytosis (HLH), EBV infection and recurrent splenomegaly (178). However, patients with XIAP deficiency may also have Crohn’s disease (4% ~20%) (179, 180). In some patients, Crohn’s disease-like enteritis is found to be the first or only clinical manifestation, with the characteristics of early age of onset, severe illness and unresponsiveness to standard treatment, including biological treatment (180–182). Interestingly, missense mutations of XIAP are mainly concentrated in two domains, BIR2 and RING, which are the key to XIAP-mediated NOD signaling pathway (183). Therefore, XIAP deficiency is considered to be the Mendelian cause of IBD. Unlike XIAP, the requirements of cIAP1/2 for the NOD signaling pathway are controversial. An experimental study performed by Bertrand et al. showed macrophages derived from *ciap1^-/^* ^–^ or *ciap2^-/-^* mice, or human colonocytes depleted cIAP1 or cIAP2 through RNAi were defective in mediating NOD signaling pathways characterized by a sharp decrease in the production of NOD-dependent pro-inflammatory cytokines and chemokine. And this blunted inflammatory response was also observed *in vivo* when *ciap1^-/–^* or *ciap2^-/-^* mice were stimulated by NOD agonists (109). Nevertheless, an *in vitro* study by Damgaard et al. found that chemical depletion of cIAP1/2 in cells *via* Smac mimetic compounds (leading to rapid degradation of cIAP1/2 without affecting the stability of XIAP) did not block NOD-mediated NF-κB activation (184). Furthermore, Stafford et al. showed that cIAP1 and cIAP2 were dispensable for NOD signaling pathway using cIAP1- or cIAP2-deficient mice *in vivo (* 110). Recently, Goncharov et al. showed that XIAP-selective antagonists (which do not affect the stability of cIAP1) can block NOD2 signalling by interfering with the binding of XIAP to RIP2, further suggesting that XIAP may be the most critical IAP required for NOD signalling (185). This discrepancy may, on the one hand, be due to the fact that these authors used different strains of cIAP-deficient mice and, on the other hand, may be due to the fact that the protein’s physiological role may differ from its role in the controlled environment of biochemical experiments. Of note, recent research has shown that cIAP1 plays a more important role in the regulation of TNF-α induced IECs death (111). In this study, compared with *ciap2^-/^* ^–^, *Xiap^-/^* ^-^mice and wild-type mice, *ciap1^-/-^* mice showed more intestinal epithelial cell apoptosis when injected with TNF-α (111). *In vitro* studies also found that intestinal epithelial cells derived from *ciap1^-/-^* mice were more sensitive to TNF-induced apoptosis (111). However, whether the specific mechanism of cIAP1 regulating the sensitivity of IECs to TNF-a depends on the its E3 ubiquitin ligase activity remains to be further studied. Moreover, cIAP2 were found to be up-regulated in colonic epithelial cells of patients with UC (112, 113). However, research on the role of cIAP2 in colitis and CAC reported inconsistent results. The *ciap2^-/-^* mice showed increased susceptibility to DSS induced acute and chronic colitis, but were resistant to AOM/DSS induced CAC (114). The effects of both inhibition of gut inflammation and promotion of tumorigenesis appear contradictory, but one possible explanation is that cIAP2 protects the IECs from inflammatory damage and promotes cell proliferation during the recovery stage of inflammation, but its expression in the CAC microenvironment promotes tumorigenesis by maintaining cancer cells survival (114).

Similarly, the role of F-box/WD repeat containing protein 7 (FBW7) in the pathogenesis of IBD also appears contradictory. FBW7 is a substrate recognition component of the SCF ubiquitin ligase, and can ubiquitinate and degrade a variety of proteins that play a role in differentiation and proliferation, including c-Jun, c-Myc, Notch and Cyclin E1, among others (186, 187). In the DSS-induced colitis mouse model, specific knockout FBW7 in IECs led to NF-κB pathway activation and exacerbated intestinal inflammation, suggesting that FBW7 may play a protective role in IBD (116). In contrast, one study by Meng et al. showed increased FBW7 expression in IECs of IBD patients and TNBS-induced colitis mice (117). In this study up-regulation of FBW7 in IECs was linked to the severity of colonic mucosal inflammation in IBD patients (117). Further molecular mechanistic studies revealed that up-regulated FBW7 activated the NF-κB signaling pathway by mediating ubiquitination degradation of IκBα, suggesting a pro-inflammatory effect for FBW7 (117). Although different colitis mouse models were used in the two studies and might account for this discrepancy, a more plausible explanation is that FBW7 homeostasis in IECs is critical in regulating intestinal inflammation, and too much or little might aggravate intestinal inflammation. Notably, another study also found that increased expression of FBW7 in IBD patients and in the DSS-induced colitis mouse model, and this expression level was significantly correlated with the severity of IBD, further suggesting that FBW7 up-regulation may be an intermediate or pathogenic factor in the pathogenesis of IBD (118). In the same study, the authors engineered mice with myeloid cell-specific FBW7 knockout and found that FBW7 deficiency in macrophages attenuated experimental colitis induced by DSS and TNBS (118). This effect was mainly attributed to FBW7 promoting K48-linked polyubiquitination and proteasome degradation of enhancer of zeste homolog 2 (EZH2) in CX3RC1^hi^ macrophages, inhibiting H3K27me3 modification of EZH2, enhancing the expression of CCL2 and CCL7, and therefore promoting the recruitment of CXCR1^int^ proinflammatory macrophages to inflamed colon tissues (118). These results suggest that, unlike the role of FBW7 in intestinal epithelial cells, FBW7 in myeloid cells has a pro-inflammatory effect. In addition, Pellino3, an important intermediate signal protein in the innate immune response pathway, was found to be down-regulated in the colon of patients with CD. Mechanistic studies revealed that Pellino3 in macrophages mediated RIPK2 ubiquitination promotes NOD2 signal transduction and plays a protective role in colitis (119). Recently, Hrd1, an E3 ubiquitin-ligase responsible for the degradation of unfolded proteins in the endoplasmic reticulum, has been found to be reduced in the intestinal tissues of IBD patients, DSS and TNBS-induced mice, as well as in the lipopolysaccharide (LPS)-induced intestinal epithelial inflammation model (120, 121). The specific molecular mechanisms underlying these phenotypes remain unclear and need further investigation.

### 3.2 Role of HECT E3s in IBD

The HECT-E3 ubiquitin ligase family was discovered through the identification of the E6AP protein, encoded by UBE3A gene. To date, 28 members of the HECT-E3 family have been identified in the human genome, and these can be divided into the NEDD4 subfamily, the HERC subfamily, and the “other” subfamily. HECT-E3 ligases plays a key regulatory role in cell fate determination, hence the reason for the association between abnormal expression or function of HECTE3 ubiquitin ligase and IBD reported in recent years. For example, HERC2 was found to be a susceptibility gene for UC (121, 188). In addition, UBR5, which is another HECTE3 ubiquitin ligase, was reported to interact with tetratricopeptide repeat domain 7A (TTC7A) and be associated with VEOIBD (189). Meanwhile, mutations in TTC7A are a pivotal pathogenic event in VEOIBD (190). Furthermore, Itch knockout mice developed spontaneous colitis and displayed increased susceptibility to DSS-induced colitis. On the other hand, Itch in the Th17 cells, innate lymphoid cells and γδT cells is able inhibit IL-17-mediated colonic inflammation and inflammation-related tumors by mediating ubiquitin degradation of retinoic acid receptor-related orphan receptor (ROR)γt (122). RORγt is a characteristic transcription factor of Th17 cells and regulates the expression of IL-17 (123). Subsequently, Itch was shown to be involved in the negative regulation of intestinal fibrosis, a common complication of IBD. In brief, the expression level of profibrotic type I collagen and α-SMA were up-regulated in Itch^-/-^ myofibroblasts under IL-17 stimulation (124). Mechanistic studies revealed that Itch can bind directly to hydrogen peroxide-inducible clone-5 (HIC-5) and target it for K63-linked ubiquitination to inhibit IL-17-driven intestinal fibrosis (124). However, recent research reported alterations of the intestinal flora of Itch knockout mice and showed that treatment with broad-spectrum antibiotics can reduce spontaneous colitis in *Itch^-/-^* mice, suggesting that the imbalance of intestinal flora may have caused spontaneous colitis in *Itch^-/-^* mice (125). Moreover, tumor suppressor HACE1 deficient mice were also highly sensitive to DSS-induced experimental colitis, likely because lack of HACE1 in IECs led to reduction of TRAF2 ubiquitin and overactivation of TNF-α-induced necrosis, and subsequent inflammation (126).

Overall, we still lack sufficient information on HECT E3 ubiquitin ligases in the context of IBD. In the future, animal research using transgenic mice and functional research to identify more substrate proteins will help to clarify the role of these ligases in the occurrence and development of inflammatory bowel disease.

### 3.3 Role of RBR E3s in IBD

HOIL-1-interacting protein (HOIP) and heme-oxidized irp2 ub ligase-1 (HOIL-1), both are RBR E3 ubiquitin ligases, together with shank-associated rh domain-interacting protein (SHARPIN), form LUBCA (191). LUBAC is the only E3 ubiquitin ligase complex in mammals that can generate linear ubiquitin chains(M1-linked) *de novo*. LUBAC is indispensable for the activation of the NF-κB signaling pathway owing to its ability to mediate M1-linked ubiquitinated modifications of NEMO(also called IKKγ, part of the IKK complex), RIPK1 and RIPK2 (192). HOIP is the catalytic subunit of LUBAC, whereas HOIL-1 and SHARPIN are essential for deregulating HOIP self-inhibition and stabilizing LUBAC (191). In recent years, the important physiological roles of LUBAC and linear ubiquitin chains have been illustrated by the discoveries of various human diseases. Patients with HOIP mutations manifested symptoms such as spontaneous inflammation of multiple organs and recurrent viral and bacterial infections (193). Similarly, patients with HOIL-1 mutations exhibited symptoms such as immunodeficiency and IBD-like symptoms (194, 195). However, SHARPIN mutations have not yet been identified in patients. Notably, Activation of NF-κB in fibroblasts and B cells from HOIL-1 mutant patients is impaired, as is the recruitment of NEMO to TNFR1 signaling complex (TNFR-SC) (194). Interestingly, monocytes of HOIL-1 mutant patients were found to be highly responsive to IL-1β stimulation, which may be associated with spontaneous inflammation in patients (194). Furthermore, HOIP and HOIL-1 knockout in mice are embryo-lethal, whereas SHARPIN deficiency in mice manifests as early onset chronic proliferative dermatitis and multi-organ inflammation (196).

Additionally, variant of ariadne RBR E3 ubiquitin protein ligase 2 (ARIH2) was identified to be associated with an increased risk of IBD (197). Overall, direct evidences for the involvement of RBR E3s in the pathogenesis of IBD are still lacking, but this is exactly where researchers should focus on and make a breakthrough.

## 4 DUBs And IBD

### 4.1 The Role of USPs

Ubiquitin-specific proteases (USPs) are the largest family of DUBs, and play a regulatory role in cell cycle, signal transduction, DNA damage repair, chromosome translocation, gene transcription, autophagy, endocytosis, and apoptosis through the regulation of their substrate proteins. USPs are characterized by the presence of two conserved modes in the catalytic domain, and cysteine and histidine boxes, which include the key residues of catalysis (198). To date, 53 USP genes have been identified in the human genome and 54 in the mouse genome. In recent years, research has been carried out to explore the effect of USP family members, especially cylindromatosis (CYLD), on the pathogenesis of IBD. Findings have shown a potential role for USPs in regulating intestinal immunity and inflammation.

#### 4.1.1 CYLD

CYLD encodes one deubiquitinase of the USP family. The N-terminal of this enzyme contains three Cap-Gly domains (the first two are responsible for binding to microtubules, and the third is responsible for binding to IκB kinase IKK adaptor protein NEMO) and two proline-rich motifs. The C-terminal contains a catalytic USP domain that preferentially recognizes the polyubiquitination linked by K63 and M1 (**Figure 4**) (199). Of note, only the two DUBs, CYLD and OTU deubiquitinase with linear linkage specificity (OTULIN) can remove LUBAC mediated -M1-linked polyubiquitin chains on proteins. CYLD was initially considered to be a typical recessive tumor suppressor gene, because the mutations of this gene were linked to skin adnexal tumors in humans (200). However, CYLD has also been found to be an important negative regulator of the NF-κB signaling pathway (201), and NF-κB activation has a strong pro-inflammatory effect in IBD. Therefore, the role of CYLD in inflammatory bowel disease has also received attention from researchers.

As early as in 2005, Costello et al. found that CYLD was significantly down-regulated in the intestinal mucosal tissues of IBD patients based on genome-wide cDNA microarray data (202). Subsequently, Zhang et al. observed that CYLD-deficient mice were more susceptible to DSS-induced colonic inflammation, due to impaired negative regulation of the NF-KB signaling pathway in the B cells, T cells, and myeloid cells, and also displayed significantly increased incidence of colonic tumors compared with the control group (134). In addition, Patrick et al. found that CYLD’s deubiquitase catalytic activity was necessary for the necrosis of colonic epithelial cells and the occurrence of colitis in *FADD^IEC-KO^* mice (135). FADD is an adaptor protein required for death receptor-induced apoptosis. Colonic epithelial cell necrosis and colitis were observed to occur spontaneously following IEC specific knockout of FADD in mice (135). Moreover, Cleynen et al. conducted a large multicenter GWAS in CD and UC patients, and found that SNPs of the CYLD gene were significantly associated with CD, among which the rs12324931 was the strongest SNP (203). Importantly, Demetrios et al. investigated the role of the deubiquitination enzyme activity of CYLD in colitis -associated colorectal cancer using a conditioned CYLD inactivation mouse model (*IEC-CYLD^Δ9^* mice, which harbors a mutation that eliminated the CYLD deubiquitination domain in IECs) (136). Their study found that *IEC-CYLD ^Δ9^* mice did not exhibit spontaneous intestinal lesions before the age of 1 year, but showed an incidence of colon tumors was significantly higher than in WT mice under the pressure of AOM/DSS (136). These results suggested that the deubiquitinase activity of CYLD in IECs plays an important inhibitory role in the process of colitis-associated carcinogenesis. Furthermore, the research results from Yilang et al. revealed a new physiological role of the CYLD spliceosome sCYLD in regulating intestinal inflammation *via* TGF-β signaling (137). Their data showed that expression of spliceosome of CYLD (sCYLD) and Smad7 in the colonic mucosa lamina propria T cells of CD patients was increased and correlated with disease severity. Mice overexpressing scyld and Smad7 (*scyld/smad7* mice) developed severe spontaneous colitis caused by overactivation of effector T cells due to impaired Treg inhibition resulting from alteration of TGF-β signaling (137). In this model, sCYLD in CD4^+^T cells could not mediate deubiquitination of Smad7 linked by K63, and enhance the nuclear translocation of Smad7 and forming complex with Smad7 in the nucleus, which impaired the DNA-binding ability of Smad3 and thus negatively regulated Smad-dependent TGF-β signaling (137). Research from by Sandip et al. *f*urther clarified the anti-inflammatory mechanism of CYLD (138). Their research discovered that CYLD expression in the colonic mucosal of UC patients was decreased and negatively correlated with IL-18 abundance (138). In line with the studies described above (134), CYLD knockout caused increased destruction of intestinal epithelia and severe colonic inflammation when challenged by pathological factors. Mechanistically, CYLD-mediated K63-linked deubiquitination of NLRP6 negatively regulated NLRP6-ASC-dependent inflammasome activation and IL-18 production in IECs, thereby inhibiting intestinal inflammation (138).

#### 4.1.2 Other USP Family Members

In addition to research on CYLD, studies of other USP family members in the context of IBD also show good progress. Firstly, studies have reported that SNPs in USP3, USP25, and USP40 were associated with IBD (203, 204). In addition, based research using whole genome sequencing data from Chinese patients, the mutation frequency of USP48 gene was significantly different between ulcerative colitis-associated colorectal cancer and scattered colorectal cancer, suggesting that a potential role for this mutation in the transition from inflammation to cancer (139). Secondly, T cell-specific USP8-deficient mice spontaneously developed colitis, likely due to imbalance of T cell homeostasis, increase of intestinal CD8^+^T cells, and impaired immunosuppression of Tregs (205). These results indicate that USP8 may have a function in maintaining intestinal homeostasis by regulating T cell homeostasis. Besides, USP9X negatively regulated c-Myc by directly stabilizing FBW7 to restore damaged intestinal epithelium and inhibit the development of colitis-associated colon cancer in animal models (140). Besides, USP16 was observed to be up-regulated in colonic macrophages of IBD patients and the deletion of USP16 in macrophages alleviated DSS-induced colitis and inflammation-associated colon carcinogenesis (141). Mechanistically, USP16 selectively removed the K33-linked polyubiquitin chains from IKKβ thereby facilitating the interaction of IKKβ with p105 and phosphorylating p105, thus activating the NF-κB signaling pathway (141). Similarly, a recent study showed that USP22 exerted an antitumor effect in colorectal cancer by reducing mTOR activity (142). Subsequently, specific knockout UPS22 in IECs increased the severity of inflammation in mice with DSS colitis and promoted colitis-related colorectal cancer, further confirming the role of USP22 in repressing intestinal inflammation and tumors (143). Finally, a recent study found an increased susceptibility to DSS-induced colitis in USP38 KO mice, accompanied by higher levels of IL-6 and IL-23A in colon tissue and peripheral blood (144). Mechanistic studies uncovered a role for USP38 in stabilizing the lysine demethylase 5B (KDM5B) by removing the K48-linked polyubiquitin chains, thus promoting KDM5B-mediated histone demethylation to inhibit the expression of IL-6 and IL-23A in bone marrow-derived cells, and ultimately inhibiting the occurrence and progression of intestinal inflammation (144).

### 4.2 The Role of OTUs

Ovarian tumor-related proteases (OTUs), are the second largest family of DUBs and are important regulators of cell signaling cascades, such as NF-κB signaling, interferon signaling (206, 207). Almost all OTUs contain an OTU catalytic domain and a ubiquitin interaction domain, such as the ubiquitin interaction primitive (UIM) domain, ubiquitin related (UBA) domain, or Zinc finger (ZnF) domain. In the human genome, at least 18 genes contain an OTU domain, 14 of which have been annotated as active DUBs (208), including A20, which we will review in detail in the next section.

#### 4.2.1 A20

Among all the deubiquitinating enzymes, A20 is by far the most intensively studied in the pathogenesis of IBD. A20, also known as TNFAIP3 (TNF-α-induced protein 3), was originally thought to be a protein that protected cells from TNF-α-induced cytotoxicity (209). However, accumulating evidence showed that A20 was not only an inhibitor of TNF-α -dependent NF-κB activation, but also a negative regulator of IL-1, PRRs, and T- and B-cell antigen-induced NF-κB activation (210–212). Therefore, A20 is widely believed to exhibit anti-inflammatory properties, which are generally attributed to its role as a ubiquitin-regulating enzyme with E3 ubiquitin ligase and deubiquitinase activity (213). Structurally, A20 contains one N-terminal OTU domain and seven C-terminal ZnF domains. The OTU domain is mainly responsible for the A20 deubiquitination enzyme activity and can specifically catalyze the hydrolysis of K11-, K48- and K63- linked ubiquitination chains (214). The ZnF domain mainly mediates the E3 ubiquitin ligase activity of A20; more specifically, the ZnF4 domain of A20 has a high affinity for K63 ubiquitin, and the ZnF7 domain is able to bind to the M1 chain in an efficient manner (**Figure 4**) (214).

Dysregulation or dysfunction of A20 expression is associated with several autoimmune diseases and cancers. SNPs at the A20 locus increase susceptibility to a number of human autoimmune diseases, including IBD, type I diabetes, rheumatoid arthritis, and systemic lupus erythematosus (215–218). In the context of IBD, an earlier genome-wide scan of the IBD family showed that the occurrence of this disease was associated with mutations in the chromosome 6q region, which contained the A20 locus (219). In addition, GWAS further suggested that A20 is a susceptibility gene for CD (145, 220). More recently, a case report found novel three heterozygous A20 mutations associated with VIOIBD (221). Importantly, a number of A20 SNPs were located in the upstream, downstream or intron regions of the A20 coding region, implying that they may affect the regulatory elements or conformation of A20 expression, and may therefore affect A20 expression or function. For example, it has been found that A125V mutation may lead to conformational changes in A20 that impaired its ability to deubiquitinate and degrade the target protein TRAF2 (222). Furthermore, recent studies have shown that a A20 gene polymorphism was correlated with the efficacy of anti-TNF therapy in IBD patients (223, 224), indicating suggesting a link between abnormal A20 expression and immune activity. However, most of disease-related SNPs within the A20 locus are located in the non-coding region, and it remains unclear how they affect A20 expression and IBD pathogenesis. Therefore, A20 expression in IBD patients has the target of several research studies. Zheng et al. found that A20 expression level, at both mRNA and protein level, in the intestinal tissues of children with IBD was down-regulated either in active stage or in remission stage compared with intestinal mucosa in children without IBD (146). In a related study, Deenaz et al. found that A20 mRNA expression was increased but protein expression was lower in children with CD compared with UC and non-IBD patients (147). This difference may be due to the different experimental detection methods used, since immunohistochemistry used in the study by Zheng et al. and enzyme-linked immunosorbent assay (ELISA) in the study by Deenaz et al. These differences between mRNA and protein expression were also seen in adult IBD patients. Indeed, studies have found that while A20 mRNA expression in UC patients is significantly up-regulated and negatively associated with disease activity, its protein expression level is down-regulated (148). In addition, Garcia et al. reported that A20 expression level in the inflamed intestinal tissues of IBD patients was increased and was related to increased apoptosis of IECs (149). Regardless, it was indisputable that A20 expression is dysregulated in IBD patients, suggesting the involvement of this protein in the pathogenesis of IBD.

Many studies in recent years investigated how A20 affected the pathogenesis of IBD in different cell types. Firstly, A20 knockout mice provided important knowledge regarding biological function of A20. A20 knockout mice die prematurely due to spontaneous multiorgan inflammation and cachexia (150). A20 knockout mouse was are unable to terminate TNF-α -induced NF-κB activation, suggesting serious deficiencies in the management of inflammatory responses. Subsequently, experiments based on *A20^-/^* ^-^ mice and *A20 Tg* mice (overexpression A20) showed that A20 maintained the intestinal barrier function and supported the tight junctions of IECs by deubiquitinating non-k48 linked polyubiquitinated of occludin (151). Mice where A20 has been specifically knockout in IECs did not develop spontaneous intestinal inflammation, but showed increased sensitivity to DSS-induced colitis and hindered recovery from acute DSS-induced intestinal inflammation, suggesting a role for A20 in alleviating intestinal inflammation and in the recovery following intestinal epithelial injury (152). In addition, Kattah et al. found that mice with IECs depleted of A20 or A20-binding inhibitor of NF-κB-1 (ABIN-1) alone appeared normal, while deletion of A20 and ABIN-1 together resulted in rapid death of IECs (153). These observations suggested that A20 and ABIN-1 have a synergistic effect in maintaining the survival of IECs. Findings derived from mechanistic studies revealed that, on the one hand, A20 and ABIN-1 jointly prevented IEC death by restraining caspase8 activation and apoptosis signal transduction (153). On the other hand, A20 and ABIN-1 may also regulate different ubiquitination events of RIPK1, which affect RIPK1 phosphorylation and RIPK1 kinase activity, repressing RIPK1-mediated necrosis (153). The synergistic effect between A20 and its chaperone factor ABIN-1 may be a manifestation of genetic epistasis, that is, expression of ABIN-1 increased in the absence of A20 to compensate for A20 deficiency and the resulting decreased cell survival. Similarly, ABIN-3 has also been reported to negatively regulate intestinal inflammation caused by necrosis; this effect appears to be *via* recruitments of A20 into the TNF-RSC and cooperation with deubiquitination enzyme A20 to limit RIPK3’s ubiquitination in IBD (154).

Overexpression of A20 has also been found to sensitize IECs and intestinal organoids to TNF-α -induced apoptosis (149). Under the stimulation of TNF-α, A20 overexpression in IECs resulted in the formation of more A20 dimers, which enhanced Ripoptosome complex assembly and RIPK1-dependent apoptosis through the ZnF7 binding to linear ubiquitin (149). Therefore, a balance of A20 expression in IECs is important to protect these cells against the challenge of pathological factors. Spontaneous intestinal inflammation, characterized by loss of Paneth and goblet cells, IEC proliferation, and crypt apoptosis, have been reported in the double-knockout A20/ATG16L1 mice (155). In this study, A20 and ATG16L1 were found to reduce the expression level of each other through their OTU domain and WD40 domain, respectively (155). This post-transcriptional cross-regulation may be a novel and important control mechanism in intestinal homeostasis.

In addition to the role of A20 in IECs, the role of A20 in immune cells and in the pathogenesis of IBD is also a hot topic of research. Mice with dendritic cells-specific A20 deficiency spontaneously exhibited DC activation and amplification of activated T cells (145). In addition, the *A20^fl/fl^-Myd88^fl/fl^* double knockout mice showed the ability of A20 to inhibit MyD88 signaling and the production of pro-inflammatory cytokines in dendritic cells to maintain the homeostasis of myeloid cells and T cells (145). Furthermore, *A20^fl/fl^ CD11c-Cre* mice developed inflammatory bowel disease at 5 months of age and exhibited increased colon diameter, expanded immune cells in lamina propria, and reduced goblet cells, suggesting that DC cells require A20 to maintain intestinal immune homeostasis and limit colitis induced by epithelial damage (145). Moreover, the absence of colitis in *A20^fl/fl^ Cd11c-Cre Rag1* mice indicate that the absence of A20 in dendritic cells may led to the overactivation of intestinal T cells in *A20^fl/fl^-CD11c-Cre* mice, which may lead to IBD. Notably, *A20^fl/fl^ CD11c-Cre* mice developed arthritis spontaneously, with a pathology similar to that of IBD-related arthritis in humans. Similarly, research by Vereecke et al. did not find spontaneous intestinal inflammation in A20^IEC-KO^ and A20^Myeol-KO^ mice. However, A20 ^IEC-KO^ mice were more susceptible to experimental colitis and had increased IEC apoptosis, while *A20^Myeol-KO^* mice developed rheumatoid arthritis-like symptoms due to excessive activation of myeloid cells to produce high levels of pro-inflammatory cytokines, including TNF-α/IFN-γ (223). Therefore, *A20^IEC/Myeol-KO^* mice not only produced high levels of pro-inflammatory cytokines, but also developed spontaneous enteritis characterized by loss of Paneth and goblet cells, increased apoptosis and proliferation of IECs, and intestinal microbiota imbalance (223). Interestingly, the abundance of intestinal flora of *A20^IEC-KO^* was not altered, but the abundance of gut microbiota of *A20^Myeol-KO^* and *A20^IEC/Myeol-KO^* were significantly decreased, suggesting that A20 may act in myeloid cells to regulate gut microbiota homeostasis. Subsequent studies have shown that, before the onset of spontaneous intestinal inflammation in mice with A20 deficiency in dendritic cells, microbial homeostasis was found to be altered in a lymphocyte-independent manner, resulting in a decrease in α diversity of intestinal flora (156). Dendritic cells-specific A20 knockout expressed higher levels of antimicrobial molecules (e.g., Reg3β, Reg3γ, Pla2g2) in the ileum or proximal colon tissues, suggesting a role for A20 in these cells in limiting antimicrobial peptide expression *in vivo* and maintaining symbiotic homeostasis. Recently, A20 in macrophages has been shown to have a negative regulatory effect on DSS-induced colitis (157). A20 deficiency in myeloid cells did not affect macrophage development in the bone marrow, but A20 deficiency in macrophages contributed to increased expression of pro-inflammatory cytokines and overactivation of the NF-κB signaling pathway, thereby causing severe DSS-induced colitis (157). In general, the function of A20 in IECs seem to help maintain intestinal barrier stability by preventing cytokine induced apoptosis, while the function of A20 in immune cells seems to prevent excessive cytokines production in myeloid-derived cells.

In addition to the above-mentioned cell type-specifically knockouts of A20, Lu et al. constructed mice with A20^OTU^ and A20^ZnF4^ mutations from the OUT domain and zinc finger domain of A20 (158). Their study found that both A20 mutated mice exhibited DSS-induced colitis, suggesting that the OTU and ZnF4 domains of A20 had a role in inflammation inhibition. Mechanistic studies further indicated that the OTU domain of A20 restricted the deubiquitination of RIPK1 at K48 and K63, while the ZnF4 domain was essential for the recruitment of A20 to ubiquitination of RIPK1, and that only the presence of both could regulate RIPK1 ubiquitination and NF-κB signal transduction. Moreover, Arne et al. recently developed *A20^ZnF4ZnF7/ZnF4ZnF^* ^7^mice with both the K63 polyubiquitin-binding ZnF4 and M1 polyubiquitin-binding ZnF7 domains inactivated (159). Surprisingly, *A20^ZnF4ZnF7/ZnF4ZnF7^* mice are phenotypically similar to A20 knockout mice: multi-organ inflammation and premature death. Then, they constructed tissue-specific ZnF4 and ZnF7 domain double inactivated mice, *Tnfaip3* ^ZnF4ZnF7/ZnF4ZnF7^ *LysM-Cre* (Myeloid-specific), *Tnfaip3* ^ZnF4ZnF7/ZnF4ZnF7^ *Vil1-Cre* (IEC-specific). The former phenotypically similar to A20^Myeol-KO^ mice: progressive polyarthritis, higher concentrations of TNF and IL-6 in serum and that BMDM is hypersensitive to LPS stimulation. The latter phenotypically similar to A20^IEC-KO^ mice: susceptible to DSS colitis and all died in response to sublethal doses of TNF attack (159). These findings suggested that the ubiquitin-binding properties of the ZnF4 and ZnF7 domains were required for A20 to inhibit pro-inflammatory signaling.

#### 4.2.2 The Other OTU Family Members

In contrast to A20 studies, there were only sporadic reports about the role of OTU family members in IBD. Multiple mutations in OTUD1 have been linked to autoimmune diseases, suggesting a possible involvement in the pathogenesis of IBD (225). In addition, GWAS of patients with UC in Korea revealed genetic susceptibility sites that were significantly associated with UC in OTUD3 (226). Recently, OTUD5 was found to be up-regulated in the intestinal inflammatory tissues of IBD patients and TNBS-induced colitis mice, and IFN-γ was found to up-regulate OTUD5’s expression through a p38/MAPK-dependent mechanism. Up-regulation of OTUD5 further increased TNF-α production in LPMCs of IBD patients (160). These findings suggested that OTUD5 may be a member of the positive feedback loop that amplifies the abnormal inflammatory response in IBD.

## 5 Conclusion and Future Directions

In the past decade, much effort has been made to elucidate the molecular mechanisms underlying the pathogenesis of IBD. In this review, we highlighted the roles of E3 ubiquitin ligases and deubiquitinases as regulators of intestinal inflammation. Challenges by pathogenic factors in intestinal tissues may lead to dysregulation of E3 ubiquitin ligases and deubiquitinases. E3 ubiquitin ligases and deubiquitinases affect cell death, immune-related signaling pathways, transcription factors and target gene expression by mediating ubiquitination and deubiquitination of substrate proteins, respectively, and serve as important modulators in the occurrence and development of IBD. Importantly, accumulating evidence show that associations between dysregulation of E3 ubiquitin ligases and deubiquitinases with intestinal inflammation and CAC. These associations are complex and will need to be further explored in future studies.

In our opinion, future research will face the following challenges:(1) Currently, only a small subset of E3 ubiquitin ligase and deubiquitinase have been fully investigated in the pathogenesis of IBD, and more relevant members and their potential mechanisms involved in the regulation of IBD need to be discovered; (2) The correlation between E3 ubiquitin ligase and deubiquitinase SNPs, discovered by GWAS, and protein function will need to be verified in animal models; (3) Most studies have focused on the role of E3s and DUBs in IECs and immune cells; the role of E3s and DUBs in other intestinal cells, such as intestinal neurons, fibroblasts and endothelial cells, will need to be further investigated; (4) There is still a lack of E3s and DUBs specific inhibitors and agonists, which could be used in the treatment of colitis in animal models.

In conclusion, E3 ubiquitin ligase and DUBs play a key role in the regulation intestinal epithelial cell death, intestinal immunity, and intestinal flora. In the future, targeting the ubiquitin pathway may provide new opportunities for the treatment of IBD.

## Author Contributions

MZ, Q-SZ, and JN: writing original draft preparation. MZ, J-HY, and Z-YL: writing review manuscript. H-TG: editing the review manuscript. All authors contributed to the article and approved the submitted version.

## Funding

This work was supported by the National Natural Science Foundation of China (Grant no. 82070560 and 81470826), the Science Foundation from Science and Technology Department of Sichuan Province (Grant no.2019YFS0262) and 1.3.5 Project for Disciplines of Excellence, West China Hospital, Sichuan University (Grant no. ZYGD18023).

## Conflict of Interest

The authors declare that the research was conducted in the absence of any commercial or financial relationships that could be construed as a potential conflict of interest.

## Publisher’s Note

All claims expressed in this article are solely those of the authors and do not necessarily represent those of their affiliated organizations, or those of the publisher, the editors and the reviewers. Any product that may be evaluated in this article, or claim that may be made by its manufacturer, is not guaranteed or endorsed by the publisher.

## References

[1] Baumgart D Sandborn W. Crohn's Disease. Lancet (London England) (2012) 380(9853):1590–605. doi: 10.1016/s0140-6736(12)60026-9 22914295

[2] Kobayashi T Siegmund B Le Berre C Wei S Ferrante M Shen B. Ulcerative Colitis. Nat Rev Dis Primers (2020) 6(1):74. doi: 10.1038/s41572-020-0205-x 32913180

[3] Hindryckx P Vande Casteele N Novak G Khanna R D'Haens G Sandborn W. The Expanding Therapeutic Armamentarium for Inflammatory Bowel Disease: How to Choose the Right Drug[s] for Our Patients? J Crohns Colitis (2018) 12(1):105–19. doi: 10.1093/ecco-jcc/jjx117 28961959

[4] Ng S Shi H Hamidi N Underwood F Tang W Benchimol E. Worldwide Incidence and Prevalence of Inflammatory Bowel Disease in the 21st Century: A Systematic Review of Population-Based Studies. Lancet (London England) (2017) 390(10114):2769–78. doi: 10.1016/s0140-6736(17)32448-0 29050646

[5] Kaplan G Windsor J. The Four Epidemiological Stages in the Global Evolution of Inflammatory Bowel Disease. Nat Rev Gastroenterol Hepatol (2021) 18(1):56–66. doi: 10.1038/s41575-020-00360-x 33033392 PMC7542092

[6] Kaplan G Ng S. Understanding and Preventing the Global Increase of Inflammatory Bowel Disease. Gastroenterology (2017) 152(2):313–21.e2. doi: 10.1053/j.gastro.2016.10.020 27793607

[7] Zhao M Gönczi L Lakatos P. : The Burden of Inflammatory Bowel Disease in Europe in 2020. J Crohns Colitis (2021) 15(9):1573–87. doi: 10.1093/ecco-jcc/jjab029 33582812

[8] de Souza H Fiocchi C Iliopoulos D. The IBD Interactome: An Integrated View of Aetiology, Pathogenesis and Therapy. Nat Rev Gastroenterol Hepatol (2017) 14(12):739–49. doi: 10.1038/nrgastro.2017.110 28831186

[9] Graham D Xavier R. Pathway Paradigms Revealed From the Genetics of Inflammatory Bowel Disease. Nature (2020) 578(7796):527–39. doi: 10.1038/s41586-020-2025-2 PMC7871366 32103191

[10] Juyal G Negi S Sood A Gupta A Prasad P Senapati S. Genome-Wide Association Scan in North Indians Reveals Three Novel HLA-Independent Risk Loci for Ulcerative Colitis. Gut (2015) 64(4):571–9. doi: 10.1136/gutjnl-2013-306625 24837172

[11] Cleynen I Boucher G Jostins L Schumm L Zeissig S Ahmad T. Inherited Determinants of Crohn's Disease and Ulcerative Colitis Phenotypes: A Genetic Association Study. Lancet (London England) (2016) 387(10014):156–67. doi: 10.1016/s0140-6736(15)00465-1 PMC4714968 26490195

[12] Khor B Gardet A Xavier R. Genetics and Pathogenesis of Inflammatory Bowel Disease. Nature (2011) 474(7351):307–17. doi: 10.1038/nature10209 PMC3204665 21677747

[13] Sadaghian Sadabad M Regeling A de Goffau M Blokzijl T Weersma R Penders J. The ATG16L1-T300A Allele Impairs Clearance of Pathosymbionts in the Inflamed Ileal Mucosa of Crohn's Disease Patients. Gut (2015) 64(10):1546–52. doi: 10.1136/gutjnl-2014-307289 25253126

[14] Homer C Richmond A Rebert N Achkar J McDonald C. ATG16L1 and NOD2 Interact in an Autophagy-Dependent Antibacterial Pathway Implicated in Crohn's Disease Pathogenesis. Gastroenterology (2010) 139(5):1630–41, 1641.e1-2. doi: 10.1053/j.gastro.2010.07.006 20637199 PMC2967588

[15] Zheng H de la Morena M Suskind D. The Growing Need to Understand Very Early Onset Inflammatory Bowel Disease. Front Immunol (2021) 12:675186. doi: 10.3389/fimmu.2021.675186 34122435 PMC8187749

[16] Lee M Chang E. Inflammatory Bowel Diseases (IBD) and the Microbiome-Searching the Crime Scene for Clues. Gastroenterology (2021) 160(2):524–37. doi: 10.1053/j.gastro.2020.09.056 PMC8098834 33253681

[17] Kostic A Xavier R Gevers D. The Microbiome in Inflammatory Bowel Disease: Current Status and the Future Ahead. Gastroenterology (2014) 146(6):1489–99. doi: 10.1053/j.gastro.2014.02.009 PMC4034132 24560869

[18] Sokol H Leducq V Aschard H Pham H Jegou S Landman C. Fungal Microbiota Dysbiosis in IBD. Gut (2017) 66(6):1039–48. doi: 10.1136/gutjnl-2015-310746 PMC5532459 26843508

[19] Ananthakrishnan A Bernstein C Iliopoulos D Macpherson A Neurath M Ali R. Environmental Triggers in IBD: A Review of Progress and Evidence. Nat Rev Gastroenterol Hepatol (2018) 15(1):39–49. doi: 10.1038/nrgastro.2017.136 29018271

[20] Ho S Lewis J Mayer E Plevy S Chuang E Rappaport S. Challenges in IBD Research: Environmental Triggers. Inflamm Bowel Dis (2019) 25:S13–23. doi: 10.1093/ibd/izz076 PMC6787673 31095702

[21] Kayama H Okumura R Takeda K. Interaction Between the Microbiota, Epithelia, and Immune Cells in the Intestine. Annu Rev Immunol (2020) 38:23–48. doi: 10.1146/annurev-immunol-070119-115104 32340570

[22] Okumura R Takeda K. Roles of Intestinal Epithelial Cells in the Maintenance of Gut Homeostasis. Exp Mol Med (2017) 49(5):e338. doi: 10.1038/emm.2017.20 28546564 PMC5454438

[23] Odenwald M Turner J. The Intestinal Epithelial Barrier: A Therapeutic Target? Nat Rev Gastroenterol Hepatol (2017) 14(1):9–21. doi: 10.1038/nrgastro.2016.169 27848962 PMC5554468

[24] Schiavi E Smolinska S O'Mahony L. Intestinal Dendritic Cells. Curr Opin Gastroenterol (2015) 31(2):98–103. doi: 10.1097/mog.0000000000000155 25651073

[25] Bsat M Chapuy L Baba N Rubio M Panzini B Wassef R. Differential Accumulation and Function of Proinflammatory 6-Sulfo LacNAc Dendritic Cells in Lymph Node and Colon of Crohn's Versus Ulcerative Colitis Patients. J Leukoc Biol (2015) 98(4):671–81. doi: 10.1189/jlb.5A1014-509RR 26162403

[26] Fucikova J Palova-Jelinkova L Bartunkova J Spisek R. Induction of Tolerance and Immunity by Dendritic Cells: Mechanisms and Clinical Applications. Front Immunol (2019) 10:2393. doi: 10.3389/fimmu.2019.02393 31736936 PMC6830192

[27] Hart A Al-Hassi H Rigby R Bell S Emmanuel A Knight S. Characteristics of Intestinal Dendritic Cells in Inflammatory Bowel Diseases. Gastroenterology (2005) 129(1):50–65. doi: 10.1053/j.gastro.2005.05.013 16012934

[28] Seyedizade S Afshari K Bayat S Rahmani F Momtaz S Rezaei N. Current Status of M1 and M2 Macrophages Pathway as Drug Targets for Inflammatory Bowel Disease. Arch Immunol Ther Exp (2020) 68(2):10. doi: 10.1007/s00005-020-00576-4 32239308

[29] Liu H Dasgupta S Fu Y Bailey B Roy C Lightcap E. Subsets of Mononuclear Phagocytes Are Enriched in the Inflamed Colons of Patients With IBD. BMC Immunol (2019) 20(1):42. doi: 10.1186/s12865-019-0322-z 31718550 PMC6852755

[30] Yang R Liao Y Wang L He P Hu Y Yuan D. Exosomes Derived From M2b Macrophages Attenuate DSS-Induced Colitis. Front Immunol (2019) 10:2346. doi: 10.3389/fimmu.2019.02346 31749791 PMC6843072

[31] Cortés A Muñoz-Antoli C Esteban J Toledo R. Th2 and Th1 Responses: Clear and Hidden Sides of Immunity Against Intestinal Helminths. Trends Parasitol (2017) 33(9):678–93. doi: 10.1016/j.pt.2017.05.004 28566191

[32] Yadav M Stephan S Bluestone J. Peripherally Induced Tregs - Role in Immune Homeostasis and Autoimmunity. Front Immunol (2013) 4:232. doi: 10.3389/fimmu.2013.00232 23966994 PMC3736167

[33] Huang J Xu X Yang J. miRNAs Alter T Helper 17 Cell Fate in the Pathogenesis of Autoimmune Diseases. Front Immunol (2021) 12:593473. doi: 10.3389/fimmu.2021.593473 33968012 PMC8096907

[34] Kriegel C Amiji M. Oral TNF-α Gene Silencing Using a Polymeric Microsphere-Based Delivery System for the Treatment of Inflammatory Bowel Disease. J Control Release (2011) 150(1):77–86. doi: 10.1016/j.jconrel.2010.10.002 20959130 PMC3033993

[35] Neurath M. Cytokines in Inflammatory Bowel Disease. Nat Rev Immunol (2014) 14(5):329–42. doi: 10.1038/nri3661 24751956

[36] Uhlig H Coombes J Mottet C Izcue A Thompson C Fanger A. Characterization of Foxp3+CD4+CD25+ and IL-10-Secreting CD4+CD25+ T Cells During Cure of Colitis. J Immunol (Baltimore Md 1950) (2006) 177(9):5852–60. doi: 10.4049/jimmunol.177.9.5852 PMC6108413 17056509

[37] Hyun C. Molecular and Pathophysiological Links Between Metabolic Disorders and Inflammatory Bowel Diseases. Int J Mol Sci (2021) 22(17):9139. doi: 10.3390/ijms22179139 34502047 PMC8430512

[38] Poggi A Benelli R Venè R Costa D Ferrari N Tosetti F. Human Gut-Associated Natural Killer Cells in Health and Disease. Front Immunol (2019) 10:961. doi: 10.3389/fimmu.2019.00961 31130953 PMC6509241

[39] Atreya I Atreya R Neurath M. NF-kappaB in Inflammatory Bowel Disease. J Internal Med (2008) 263(6):591–6. doi: 10.1111/j.1365-2796.2008.01953.x 18479258

[40] Yu H Lin L Zhang Z Hu H. Targeting NF-κB Pathway for the Therapy of Diseases: Mechanism and Clinical Study. Signal Transduct Target Ther (2020) 5(1):209. doi: 10.1038/s41392-020-00312-6 32958760 PMC7506548

[41] Blanchett S Boal-Carvalho I Layzell S Seddon B. NF-κB and Extrinsic Cell Death Pathways - Entwined Do-Or-Die Decisions for T Cells. Trends Immunol (2021) 42(1):76–88. doi: 10.1016/j.it.2020.10.013 33246882

[42] Won M Byun H Park K Hur G. Post-Translational Control of NF-κB Signaling by Ubiquitination. Arch Pharm Res (2016) 39(8):1075–84. doi: 10.1007/s12272-016-0772-2 27287455

[43] Garcia-Carbonell R Yao S Das S Guma M. Dysregulation of Intestinal Epithelial Cell RIPK Pathways Promotes Chronic Inflammation in the IBD Gut. Front Immunol (2019) 10:1094. doi: 10.3389/fimmu.2019.01094 31164887 PMC6536010

[44] Taniguchi K Karin M. NF-κB, Inflammation, Immunity and Cancer: Coming of Age. Nat Rev Immunol (2018) 18(5):309–24. doi: 10.1038/nri.2017.142 29379212

[45] Ferrand J Ferrero R. Recognition of Extracellular Bacteria by NLRs and Its Role in the Development of Adaptive Immunity. Front Immunol (2013) 4:344. doi: 10.3389/fimmu.2013.00344 24155747 PMC3801148

[46] Verstrepen L Bekaert T Chau T Tavernier J Chariot A Beyaert R. TLR-4, IL-1R and TNF-R Signaling to NF-Kappab: Variations on a Common Theme. Cell Mol Life Sci (2008) 65(19):2964–78. doi: 10.1007/s00018-008-8064-8 PMC11131687 18535784

[47] Kawasaki T Kawai T. Toll-Like Receptor Signaling Pathways. Front Immunol (2014) 5:461. doi: 10.3389/fimmu.2014.00461 25309543 PMC4174766

[48] Lavelle E Murphy C O'Neill L Creagh E. The Role of TLRs, NLRs, and RLRs in Mucosal Innate Immunity and Homeostasis. Mucosal Immunol (2010) 3(1):17–28. doi: 10.1038/mi.2009.124 19890268 PMC3428627

[49] Bank S Skytt Andersen P Burisch J Pedersen N Roug S Galsgaard J. Polymorphisms in the Inflammatory Pathway Genes TLR2, TLR4, TLR9, LY96, NFKBIA, NFKB1, TNFA, TNFRSF1A, IL6R, IL10, IL23R, PTPN22, and PPARG Are Associated With Susceptibility of Inflammatory Bowel Disease in a Danish Cohort. PloS One (2014) 9(6):e98815. doi: 10.1371/journal.pone.0098815 24971461 PMC4074037

[50] Topal Y Gyrd-Hansen M. RIPK2 NODs to XIAP and IBD. Semin Cell Dev Biol (2021) 109:144–50. doi: 10.1016/j.semcdb.2020.07.001 32631784

[51] Cho J Abraham C. Inflammatory Bowel Disease Genetics: Nod2. Annu Rev Med (2007) 58:401–16. doi: 10.1146/annurev.med.58.061705.145024 16987083

[52] Cildir G Low K Tergaonkar V. Noncanonical NF-κB Signaling in Health and Disease. Trends Mol Med (2016) 22(5):414–29. doi: 10.1016/j.molmed.2016.03.002 27068135

[53] Sun S. The Noncanonical NF-κB Pathway. Immunol Rev (2012) 246(1):125–40. doi: 10.1111/j.1600-065X.2011.01088.x PMC3313452 22435551

[54] McDaniel D Eden K Ringel V Allen I. Emerging Roles for Noncanonical NF-κB Signaling in the Modulation of Inflammatory Bowel Disease Pathobiology. Inflamm Bowel Dis (2016) 22(9):2265–79. doi: 10.1097/mib.0000000000000858 PMC4992436 27508514

[55] Goyal N Rana A Ahlawat A Bijjem K Kumar P. Animal Models of Inflammatory Bowel Disease: A Review. Inflammopharmacology (2014) 22(4):219–33. doi: 10.1007/s10787-014-0207-y 24906689

[56] Eichele D Kharbanda K. Dextran Sodium Sulfate Colitis Murine Model: An Indispensable Tool for Advancing Our Understanding of Inflammatory Bowel Diseases Pathogenesis. World J Gastroenterol (2017) 23(33):6016–29. doi: 10.3748/wjg.v23.i33.6016 PMC5597494 28970718

[57] Zheng H Lu Z Wang R Chen N Zheng P. Establishing the Colitis-Associated Cancer Progression Mouse Models. Int J Immunopathol Pharmacol (2016) 29(4):759–63. doi: 10.1177/0394632016670919 PMC5806830 27694612

[58] Silva I Pinto R Mateus V. Preclinical Study *In Vivo* for New Pharmacological Approaches in Inflammatory Bowel Disease: A Systematic Review of Chronic Model of TNBS-Induced Colitis. J Clin Med (2019) 8(10):1574. doi: 10.3390/jcm8101574 PMC6832474 31581545

[59] Jiminez J Uwiera T Douglas Inglis G Uwiera R. Animal Models to Study Acute and Chronic Intestinal Inflammation in Mammals. Gut Pathog (2015) 7:29. doi: 10.1186/s13099-015-0076-y 26561503 PMC4641401

[60] Mizoguchi A. Animal Models of Inflammatory Bowel Disease. Prog Mol Biol Trans Sci (2012) 105:263–320. doi: 10.1016/b978-0-12-394596-9.00009-3 22137435

[61] Ostanin D Bao J Koboziev I Gray L Robinson-Jackson S Kosloski-Davidson M. T Cell Transfer Model of Chronic Colitis: Concepts, Considerations, and Tricks of the Trade. Am J Physiol Gastrointest Liver Physiol (2009) 296(2):G135–46. doi: 10.1152/ajpgi.90462.2008 PMC2643911 19033538

[62] Pastorelli L De Salvo C Mercado J Vecchi M Pizarro T. Central Role of the Gut Epithelial Barrier in the Pathogenesis of Chronic Intestinal Inflammation: Lessons Learned From Animal Models and Human Genetics. Front Immunol (2013) 4:280. doi: 10.3389/fimmu.2013.00280 24062746 PMC3775315

[63] Mizoguchi E Low D Ezaki Y Okada T. Recent Updates on the Basic Mechanisms and Pathogenesis of Inflammatory Bowel Diseases in Experimental Animal Models. Intest Res (2020) 18(2):151–67. doi: 10.5217/ir.2019.09154 PMC7206339 32326669

[64] Wilk J Bilsborough J Viney J. The Mdr1a-/- Mouse Model of Spontaneous Colitis: A Relevant and Appropriate Animal Model to Study Inflammatory Bowel Disease. Immunol Res (2005) 31(2):151–9. doi: 10.1385/ir:31:2:151 15778512

[65] Ciechanover A. The Unravelling of the Ubiquitin System. Nat Rev Mol Cell Biol (2015) 16(5):322–4. doi: 10.1038/nrm3982 25907614

[66] Zinngrebe J Montinaro A Peltzer N Walczak H. Ubiquitin in the Immune System. EMBO Rep (2014) 15(1):28–45. doi: 10.1002/embr.201338025 24375678 PMC4303447

[67] Mattern M Sutherland J Kadimisetty K Barrio R Rodriguez M. Using Ubiquitin Binders to Decipher the Ubiquitin Code. Trends Biochem Sci (2019) 44(7):599–615. doi: 10.1016/j.tibs.2019.01.011 30819414

[68] Bremm A Komander D. Emerging Roles for Lys11-Linked Polyubiquitin in Cellular Regulation. Trends Biochem Sci (2011) 36(7):355–63. doi: 10.1016/j.tibs.2011.04.004 21641804

[69] Pohl C Dikic I. Cellular Quality Control by the Ubiquitin-Proteasome System and Autophagy. Science (New York N Y) (2019) 366(6467):818–22. doi: 10.1126/science.aax3769 31727826

[70] Li J Chai Q Liu C. The Ubiquitin System: A Critical Regulator of Innate Immunity and Pathogen-Host Interactions. Cell Mol Immunol (2016) 13(5):560–76. doi: 10.1038/cmi.2016.40 PMC5037286 27524111

[71] Berndsen C Wolberger C. New Insights Into Ubiquitin E3 Ligase Mechanism. Nat Struct Mol Biol (2014) 21(4):301–7. doi: 10.1038/nsmb.2780 24699078

[72] Smit J Sixma T. RBR E3-Ligases at Work. EMBO Rep (2014) 15(2):142–54. doi: 10.1002/embr.201338166 PMC3989860 24469331

[73] Metzger M Pruneda J Klevit R Weissman A. RING-Type E3 Ligases: Master Manipulators of E2 Ubiquitin-Conjugating Enzymes and Ubiquitination. Biochim Biophys Acta (2014) 1843(1):47–60. doi: 10.1016/j.bbamcr.2013.05.026 23747565 PMC4109693

[74] Scheffner M Nuber U Huibregtse J. Protein Ubiquitination Involving an E1-E2-E3 Enzyme Ubiquitin Thioester Cascade. Nature (1995) 373(6509):81–3. doi: 10.1038/373081a0 7800044

[75] Sluimer J Distel B. Regulating the Human HECT E3 Ligases. Cell Mol Life Sci (2018) 75(17):3121–41. doi: 10.1007/s00018-018-2848-2 PMC6063350 29858610

[76] Park J Cho J Song E. Ubiquitin-Proteasome System (UPS) as a Target for Anticancer Treatment. Arch Pharm Res (2020) 43(11):1144–61. doi: 10.1007/s12272-020-01281-8 PMC7651821 33165832

[77] Piao J Hasegawa M Heissig B Hattori K Takeda K Iwakura Y. Tumor Necrosis Factor Receptor-Associated Factor (TRAF) 2 Controls Homeostasis of the Colon to Prevent Spontaneous Development of Murine Inflammatory Bowel Disease. J Biol Chem (2011) 286(20):17879–88. doi: 10.1074/jbc.M111.221853 PMC3093863 21393251

[78] Piao J Yagita H Okumura K Nakano H. Aberrant Accumulation of Interleukin-10-Secreting Neutrophils in TRAF2-Deficient Mice. Immunol Cell Biol (2012) 90(9):881–8. doi: 10.1038/icb.2012.22 22546736

[79] Jin J Xiao Y Hu H Zou Q Li Y Gao Y. Proinflammatory TLR Signalling Is Regulated by a TRAF2-Dependent Proteolysis Mechanism in Macrophages. Nat Commun (2015) 6:5930. doi: 10.1038/ncomms6930 25565375 PMC4286812

[80] Qiao Y Shen J Gu Y Tong J Xu X Huang M. Gene Expression of Tumor Necrosis Factor Receptor Associated-Factor (TRAF)-1 and TRAF-2 in Inflammatory Bowel Disease. J Dig Dis (2013) 14(5):244–50. doi: 10.1111/1751-2980.12044 23414308

[81] Shen J Qiao Y Ran Z Wang T. Up-Regulation and Pre-Activation of TRAF3 and TRAF5 in Inflammatory Bowel Disease. Int J Med Sci (2013) 10(2):156–63. doi: 10.7150/ijms.5457 PMC3547213 23329887

[82] Ma C Lin W Liu Z Tang W Gautam R Li H. NDR1 Protein Kinase Promotes IL-17- and TNF-α-Mediated Inflammation by Competitively Binding TRAF3. EMBO Rep (2017) 18(4):586–602. doi: 10.15252/embr.201642140 28219902 PMC5376972

[83] Shen J Qiao Y Ran Z Wang T. Different Activation of TRAF4 and TRAF6 in Inflammatory Bowel Disease. Mediators Inflamm (2013) 2013:647936. doi: 10.1155/2013/647936 23431243 PMC3569908

[84] Shang J Li L Wang X Pan H Liu S He R. Disruption of Tumor Necrosis Factor Receptor-Associated Factor 5 Exacerbates Murine Experimental Colitis *via* Regulating T Helper Cell-Mediated Inflammation. Mediators Inflamm (2016) 2016:9453745. doi: 10.1155/2016/9453745 27110068 PMC4823516

[85] Vlantis K Polykratis A Welz P van Loo G Pasparakis M Wullaert A. TLR-Independent Anti-Inflammatory Function of Intestinal Epithelial TRAF6 Signalling Prevents DSS-Induced Colitis in Mice. Gut (2016) 65(6):935–43. doi: 10.1136/gutjnl-2014-308323 PMC4893119 25761602

[86] Chen M Zhao Z Meng Q Liang P Su Z Wu Y. TRIM14 Promotes Noncanonical NF-κB Activation by Modulating P100/P52 Stability *via* Selective Autophagy. Adv Sci (Weinh Baden-Wurttemberg Germany) (2020) 7(1):1901261. doi: 10.1002/advs.201901261 PMC6947505 31921549

[87] Zhou G Wu W Yu L Yu T Yang W Wang P. Tripartite Motif-Containing (TRIM) 21 Negatively Regulates Intestinal Mucosal Inflammation Through Inhibiting T1/T17 Cell Differentiation in Patients With Inflammatory Bowel Diseases. J Allergy Clin Immunol (2018) 142(4):1218–28.e12. doi: 10.1016/j.jaci.2017.09.038 29113905

[88] Zhou G Wu H Lin J Lin R Feng B Liu Z. TRIM21 Is Decreased in Colitis-Associated Cancer and Negatively Regulates Epithelial Carcinogenesis. Inflamm Bowel Dis (2021) 27(4):458–68. doi: 10.1093/ibd/izaa229 32860065

[89] Zhao J Cai B Shao Z Zhang L Zheng Y Ma C. TRIM26 Positively Regulates the Inflammatory Immune Response Through K11-Linked Ubiquitination of TAB1. Cell Death Differ (2021) 28(11):3077–91. doi: 10.1038/s41418-021-00803-1 PMC8563735 34017102

[90] Zurek B Schoultz I Neerincx A Napolitano L Birkner K Bennek E. TRIM27 Negatively Regulates NOD2 by Ubiquitination and Proteasomal Degradation. PloS One (2012) 7(7):e41255. doi: 10.1371/journal.pone.0041255 22829933 PMC3400628

[91] Ohya S Fukuyo Y Kito H Shibaoka R Matsui M Niguma H. Upregulation of KCa3.1 K(+) Channel in Mesenteric Lymph Node CD4(+) T Lymphocytes From a Mouse Model of Dextran Sodium Sulfate-Induced Inflammatory Bowel Disease. Am J Physiol Gastrointest Liver Physiol (2014) 306(10):G873–85. doi: 10.1152/ajpgi.00156.2013 24674776

[92] Zaman M Shinagawa T Ishii S. Trim27-Deficient Mice Are Susceptible to Streptozotocin-Induced Diabetes. FEBS Open Bio (2013) 4:60–4. doi: 10.1016/j.fob.2013.12.002 PMC3879403 24392305

[93] Zhang H Xu Z Lin H Li M Xia T Cui K. TRIM27 Mediates STAT3 Activation at Retromer-Positive Structures to Promote Colitis and Colitis-Associated Carcinogenesis. Nat Commun (2018) 9(1):3441. doi: 10.1038/s41467-018-05796-z 30143645 PMC6109048

[94] Song H Liu B Huai W Yu Z Wang W Zhao J. The E3 Ubiquitin Ligase TRIM31 Attenuates NLRP3 Inflammasome Activation by Promoting Proteasomal Degradation of NLRP3. Nat Commun (2016) 7:13727. doi: 10.1038/ncomms13727 27929086 PMC5155141

[95] Petit V Parcelier A Mathé C Barroca V Torres C Lewandowski D. TRIM33 Deficiency in Monocytes and Macrophages Impairs Resolution of Colonic Inflammation. EBioMedicine (2019) 44:60–70. doi: 10.1016/j.ebiom.2019.05.037 31130476 PMC6604767

[96] Lian Q Yan S Yin Q Yan C Zheng W Gu W. TRIM34 Attenuates Colon Inflammation and Tumorigenesis by Sustaining Barrier Integrity. Cell Mol Immunol (2021) 18(2):350–62. doi: 10.1038/s41423-020-0366-2 PMC8027410 32094504

[97] Eyking A Ferber F Köhler S Reis H Cario E. TRIM58 Restrains Intestinal Mucosal Inflammation by Negatively Regulating TLR2 in Myeloid Cells. J Immunol (Baltimore Md 1950) (2019) 203(6):1636–49. doi: 10.4049/jimmunol.1900413 PMC6731451 31383741

[98] Cao Z Conway K Heath R Rush J Leshchiner E Ramirez-Ortiz Z. Ubiquitin Ligase TRIM62 Regulates CARD9-Mediated Anti-Fungal Immunity and Intestinal Inflammation. Immunity (2015) 43(4):715–26. doi: 10.1016/j.immuni.2015.10.005 PMC4672733 26488816

[99] Fujita Y Khateb A Li Y Tinoco R Zhang T Bar-Yoseph H. Regulation of S100A8 Stability by RNF5 in Intestinal Epithelial Cells Determines Intestinal Inflammation and Severity of Colitis. Cell Rep (2018) 24(12):3296–3311.e6. doi: 10.1016/j.celrep.2018.08.057 30232010 PMC6185744

[100] Zhu Y Shi Y Ke X Xuan L Ma Z. RNF8 Induces Autophagy and Reduces Inflammation by Promoting AKT Degradation *via* Ubiquitination in Ulcerative Colitis Mice. J Biochem (2020) 168(5):445–53. doi: 10.1093/jb/mvaa068 32597970

[101] Tarcic O Pateras I Cooks T Shema E Kanterman J Ashkenazi H. RNF20 Links Histone H2B Ubiquitylation With Inflammation and Inflammation-Associated Cancer. Cell Rep (2016) 14(6):1462–76. doi: 10.1016/j.celrep.2016.01.020 PMC4761112 26854224

[102] Kosinsky R Chua R Qui M Saul D Mehlich D Ströbel M. Loss of RNF40 Decreases NF-κB Activity in Colorectal Cancer Cells and Reduces Colitis Burden in Mice. J Crohns Colitis (2019) 13(3):362–73. doi: 10.1093/ecco-jcc/jjy165 PMC6599279 30321325

[103] Mukai A Iijima H Hiyama S Fujii H Shinzaki S Inoue T. Regulation of Anergy-Related Ubiquitin E3 Ligase, GRAIL, in Murine Models of Colitis and Patients With Crohn's Disease. J Gastroenterol (2014) 49(12):1524–35. doi: 10.1007/s00535-013-0923-x 24356810

[104] Egawa S Iijima H Shinzaki S Nakajima S Wang J Kondo J. Upregulation of GRAIL Is Associated With Remission of Ulcerative Colitis. Am J Physiol Gastrointest Liver Physiol (2008) 295(1):G163–9. doi: 10.1152/ajpgi.90242.2008 18467499

[105] Yu Q Zhang S Chao K Feng R Wang H Li M. E3 Ubiquitin Ligase RNF183 Is a Novel Regulator in Inflammatory Bowel Disease. J Crohns Colitis (2016) 10(6):713–25. doi: 10.1093/ecco-jcc/jjw023 26818663

[106] Wu Y Kimura Y Okamoto T Matsuhisa K Asada R Saito A. Inflammatory Bowel Disease-Associated Ubiquitin Ligase RNF183 Promotes Lysosomal Degradation of DR5 and TRAIL-Induced Caspase Activation. Sci Rep (2019) 9(1):20301. doi: 10.1038/s41598-019-56748-6 31889078 PMC6937276

[107] Fujimoto K Kinoshita M Tanaka H Okuzaki D Shimada Y Kayama H. Regulation of Intestinal Homeostasis by the Ulcerative Colitis-Associated Gene RNF186. Mucosal Immunol (2017) 10(2):446–59. doi: 10.1038/mi.2016.58 27381925

[108] Ranjan K Hedl M Sinha S Zhang X Abraham C. Ubiquitination of ATF6 by Disease-Associated RNF186 Promotes the Innate Receptor-Induced Unfolded Protein Response. J Clin Invest (2021) 131(17):e145472. doi: 10.1172/jci145472 34623328 PMC8409591

[109] Bertrand M Doiron K Labbé K Korneluk R Barker P Saleh M. Cellular Inhibitors of Apoptosis Ciap1 and Ciap2 Are Required for Innate Immunity Signaling by the Pattern Recognition Receptors NOD1 and NOD2. Immunity (2009) 30(6):789–801. doi: 10.1016/j.immuni.2009.04.011 19464198

[110] Stafford C Lawlor K Heim V Bankovacki A Bernardini J Silke J. IAPs Regulate Distinct Innate Immune Pathways to Co-Ordinate the Response to Bacterial Peptidoglycans. Cell Rep (2018) 22(6):1496–508. doi: 10.1016/j.celrep.2018.01.024 29425505

[111] Grabinger T Bode K Demgenski J Seitz C Delgado M Kostadinova F. Inhibitor of Apoptosis Protein-1 Regulates Tumor Necrosis Factor-Mediated Destruction of Intestinal Epithelial Cells. Gastroenterology (2017) 152(4):867–79. doi: 10.1053/j.gastro.2016.11.019 27889570

[112] Seidelin J Vainer B Andresen L Nielsen O. Upregulation of Ciap2 in Regenerating Colonocytes in Ulcerative Colitis. Virchows Arch (2007) 451(6):1031–8. doi: 10.1007/s00428-007-0517-1 17972100

[113] Seidelin J. Regulation of Antiapoptotic and Cytoprotective Pathways in Colonic Epithelial Cells in Ulcerative Colitis. Scand J Gastroenterol (2015) 50(sup1):1–29. doi: 10.3109/00365521.2016.1101245 26513451

[114] Dagenais M Dupaul-Chicoine J Champagne C Skeldon A Morizot A Saleh M. A Critical Role for Cellular Inhibitor of Protein 2 (Ciap2) in Colitis-Associated Colorectal Cancer and Intestinal Homeostasis Mediated by the Inflammasome and Survival Pathways. Mucosal Immunol (2016) 9(1):146–58. doi: 10.1038/mi.2015.46 26037070

[115] Damgaard R Nachbur U Yabal M Wong W Fiil B Kastirr M. The Ubiquitin Ligase XIAP Recruits LUBAC for NOD2 Signaling in Inflammation and Innate Immunity. Mol Cell (2012) 46(6):746–58. doi: 10.1016/j.molcel.2012.04.014 22607974

[116] Li H Liang Y Lai X Wang W Zhang J Chen S. Genetic Deletion of Fbw7 in the Mouse Intestinal Epithelium Aggravated Dextran Sodium Sulfate-Induced Colitis by Modulating the Inflammatory Response of NF-κB Pathway. Biochem Biophys Res Commun (2018) 498(4):869–76. doi: 10.1016/j.bbrc.2018.03.072 29550488

[117] Meng Q Wu W Pei T Xue J Xiao P Sun L. miRNA-129/FBW7/NF-κB, a Novel Regulatory Pathway in Inflammatory Bowel Disease. Mol Ther Nucleic Acids (2020) 19:731–40. doi: 10.1016/j.omtn.2019.10.048 PMC6965515 31945730

[118] He J Song Y Li G Xiao P Liu Y Xue Y. Fbxw7 Increases CCL2/7 in CX3CR1hi Macrophages to Promote Intestinal Inflammation. J Clin Invest (2019) 129(9):3877–93. doi: 10.1172/jci123374 PMC6715366 31246581

[119] Yang S Wang B Humphries F Jackson R Healy M Bergin R. Pellino3 Ubiquitinates RIP2 and Mediates Nod2-Induced Signaling and Protective Effects in Colitis. Nat Immunol (2013) 14(9):927–36. doi: 10.1038/ni.2669 23892723

[120] Dong J Xia K Liang W Liu L Yang F Fang X. Ginsenoside Rb1 Alleviates Colitis in Mice *via* Activation of Endoplasmic Reticulum-Resident E3 Ubiquitin Ligase Hrd1 Signaling Pathway. Acta Pharmacol Sin (2020) 42(9):1461–71. doi: 10.1038/s41401-020-00561-9 PMC8379258 33268823

[121] Sun S Lourie R Cohen S Ji Y Goodrich J Poole A. Epithelial Sel1L Is Required for the Maintenance of Intestinal Homeostasis. Mol Biol Cell (2016) 27(3):483–90. doi: 10.1091/mbc.E15-10-0724 PMC4751599 26631554

[122] Kathania M Khare P Zeng M Cantarel B Zhang H Ueno H. Itch Inhibits IL-17-Mediated Colon Inflammation and Tumorigenesis by ROR-γt Ubiquitination. Nat Immunol (2016) 17(8):997–1004. doi: 10.1038/ni.3488 27322655

[123] Capone A Volpe E. Transcriptional Regulators of T Helper 17 Cell Differentiation in Health and Autoimmune Diseases. Front Immunol (2020) 11:348. doi: 10.3389/fimmu.2020.00348 32226427 PMC7080699

[124] Paul J Singh A Kathania M Elviche T Zeng M Basrur V. IL-17-Driven Intestinal Fibrosis Is Inhibited by Itch-Mediated Ubiquitination of HIC-5. Mucosal Immunol (2018) 11(2):427–36. doi: 10.1038/mi.2017.53 28612841

[125] Kathania M Tsakem E Theiss A Venuprasad K. Gut Microbiota Contributes to Spontaneous Colitis in E3 Ligase Itch-Deficient Mice. J Immunol (Baltimore Md 1950) (2020) 204(8):2277–84. doi: 10.4049/jimmunol.1701478 PMC7811274 32169841

[126] Tortola L Nitsch R Bertrand M Kogler M Redouane Y Kozieradzki I. The Tumor Suppressor Hace1 Is a Critical Regulator of TNFR1-Mediated Cell Fate. Cell Rep (2016) 15(7):1481–92. doi: 10.1016/j.celrep.2016.04.032 PMC4893156 27160902

[127] Mevissen T Komander D. Mechanisms of Deubiquitinase Specificity and Regulation. Annu Rev Biochem (2017) 86:159–92. doi: 10.1146/annurev-biochem-061516-044916 28498721

[128] Mennerich D Kubaichuk K Kietzmann T. DUBs, Hypoxia, and Cancer. Trends Cancer (2019) 5(10):632–53. doi: 10.1016/j.trecan.2019.08.005 31706510

[129] Li Y Reverter D. Molecular Mechanisms of DUBs Regulation in Signaling and Disease. Int J Mol Sci (2021) 22(3):986. doi: 10.3390/ijms22030986 33498168 PMC7863924

[130] Nijman S Luna-Vargas M Velds A Brummelkamp T Dirac A Sixma T. A Genomic and Functional Inventory of Deubiquitinating Enzymes. Cell (2005) 123(5):773–86. doi: 10.1016/j.cell.2005.11.007 16325574

[131] Song L Rape M. Reverse the Curse–the Role of Deubiquitination in Cell Cycle Control. Curr Opin Cell Biol (2008) 20(2):156–63. doi: 10.1016/j.ceb.2008.01.012 PMC2387050 18346885

[132] Kwon S Saindane M Baek K. P53 Stability Is Regulated by Diverse Deubiquitinating Enzymes. Biochim Biophys Acta Rev Cancer (2017) 1868(2):404–11. doi: 10.1016/j.bbcan.2017.08.001 28801249

[133] He M Zhou Z Wu G Chen Q Wan Y. Emerging Role of DUBs in Tumor Metastasis and Apoptosis: Therapeutic Implication. Pharmacol Ther (2017) 177:96–107. doi: 10.1016/j.pharmthera.2017.03.001 28279784 PMC5565705

[134] Zhang J Stirling B Temmerman S Ma C Fuss I Derry J. Impaired Regulation of NF-kappaB and Increased Susceptibility to Colitis-Associated Tumorigenesis in CYLD-Deficient Mice. J Clin Invest (2006) 116(11):3042–9. doi: 10.1172/jci28746 PMC1616194 17053834

[135] Welz P Wullaert A Vlantis K Kondylis V Fernández-Majada V Ermolaeva M. FADD Prevents RIP3-Mediated Epithelial Cell Necrosis and Chronic Intestinal Inflammation. Nature (2011) 477(7364):330–4. doi: 10.1038/nature10273 21804564

[136] Karatzas D Xanthopoulos K Kotantaki P Pseftogas A Teliousis K Hatzivassiliou E. Inactivation of CYLD in Intestinal Epithelial Cells Exacerbates Colitis-Associated Colorectal Carcinogenesis - A Short Report. Cell Oncol (Dordrecht) (2016) 39(3):287–93. doi: 10.1007/s13402-016-0279-3 PMC13001861 27042826

[137] Tang Y Reissig S Glasmacher E Regen T Wanke F Nikolaev A. Alternative Splice Forms of CYLD Mediate Ubiquitination of SMAD7 to Prevent TGFB Signaling and Promote Colitis. Gastroenterology (2019) 156(3):692–707.e7. doi: 10.1053/j.gastro.2018.10.023 30315770

[138] Mukherjee S Kumar R Tsakem Lenou E Basrur V Kontoyiannis D Ioakeimidis F. Deubiquitination of NLRP6 Inflammasome by Cyld Critically Regulates Intestinal Inflammation. Nat Immunol (2020) 21(6):626–35. doi: 10.1038/s41590-020-0681-x PMC7881443 32424362

[139] Yan P Wang Y Meng X Yang H Liu Z Qian J. Whole Exome Sequencing of Ulcerative Colitis-Associated Colorectal Cancer Based on Novel Somatic Mutations Identified in Chinese Patients. Inflamm Bowel Dis (2019) 25(8):1293–301. doi: 10.1093/ibd/izz020 30794281

[140] Khan O Carvalho J Spencer-Dene B Mitter R Frith D Snijders A. The Deubiquitinase USP9X Regulates FBW7 Stability and Suppresses Colorectal Cancer. J Clin Invest (2018) 128(4):1326–37. doi: 10.1172/jci97325 PMC5873885 29346117

[141] Yu J Huang T Zhang Y Mao X Huang L Li Y. Substrate-Specific Recognition of IKKs Mediated by USP16 Facilitates Autoimmune Inflammation. Sci Adv (2021) 7(3):eabc4009. doi: 10.1126/sciadv.abc4009 33523871 PMC7806237

[142] Kosinsky R Zerche M Saul D Wang X Wohn L Wegwitz F. USP22 Exerts Tumor-Suppressive Functions in Colorectal Cancer by Decreasing mTOR Activity. Cell Death Differ (2020) 27(4):1328–40. doi: 10.1038/s41418-019-0420-8 PMC7205880 31527800

[143] Kosinsky R Saul D Ammer-Herrmenau C Faubion W Neesse A Johnsen S. SPARCUSP22 Suppresses Expression in Acute Colitis and Inflammation-Associated Colorectal Cancer. Cancers (2021) 13(8):1817. doi: 10.3390/cancers13081817 33920268 PMC8070211

[144] Zhao Z Su Z Liang P Liu D Yang S Wu Y. USP38 Couples Histone Ubiquitination and Methylation *via* KDM5B to Resolve Inflammation. Adv Sci (Weinh Baden-Wurttemberg Germany) (2020) 7(22):2002680. doi: 10.1002/advs.202002680 PMC7675183 33240782

[145] Hammer G Turer E Taylor K Fang C Advincula R Oshima S. Expression of A20 by Dendritic Cells Preserves Immune Homeostasis and Prevents Colitis and Spondyloarthritis. Nat Immunol (2011) 12(12):1184–93. doi: 10.1038/ni.2135 PMC3419270 22019834

[146] Zheng C Huang Y. Expression of Zinc Finger Protein A20 in Pediatric Inflammatory Bowel Disease. Zhonghua Er Ke Za Zhi (2011) 49(4):261–5. doi:10.1007/s12583-011-0153-1 21624200

[147] Zaidi D Huynh H Carroll M Baksh S Wine E. Tumor Necrosis Factor α-Induced Protein 3 (A20) Is Dysregulated in Pediatric Crohn Disease. Clin Exp Gastroenterol (2018) 11:217–31. doi: 10.2147/ceg.S148217 PMC5985767 29881302

[148] Majumdar I Ahuja V Paul J. Altered Expression of Tumor Necrosis Factor Alpha -Induced Protein 3 Correlates With Disease Severity in Ulcerative Colitis. Sci Rep (2017) 7(1):9420. doi: 10.1038/s41598-017-09796-9 28842689 PMC5572729

[149] Garcia-Carbonell R Wong J Kim J Close L Boland B Wong T. Elevated A20 Promotes TNF-Induced and RIPK1-Dependent Intestinal Epithelial Cell Death. Proc Natl Acad Sci USA (2018) 115(39):E9192–200. doi: 10.1073/pnas.1810584115 PMC6166836 30209212

[150] Lee E Boone D Chai S Libby S Chien M Lodolce J. Failure to Regulate TNF-Induced NF-kappaB and Cell Death Responses in A20-Deficient Mice. Science (New York N Y) (2000) 289(5488):2350–4. doi: 10.1126/science.289.5488.2350 PMC3582399 11009421

[151] Kolodziej L Lodolce J Chang J Schneider J Grimm W Bartulis S. TNFAIP3 Maintains Intestinal Barrier Function and Supports Epithelial Cell Tight Junctions. PloS One (2011) 6(10):e26352. doi: 10.1371/journal.pone.0026352 22031828 PMC3198775

[152] Vereecke L Sze M Mc Guire C Rogiers B Chu Y Schmidt-Supprian M. Enterocyte-Specific A20 Deficiency Sensitizes to Tumor Necrosis Factor-Induced Toxicity and Experimental Colitis. J Exp Med (2010) 207(7):1513–23. doi: 10.1084/jem.20092474 PMC2901067 20530205

[153] Kattah M Shao L Rosli Y Shimizu H Whang M Advincula R. A20 and ABIN-1 Synergistically Preserve Intestinal Epithelial Cell Survival. J Exp Med (2018) 215(7):1839–52. doi: 10.1084/jem.20180198 PMC6028510 29930103

[154] Zhou M He J Shi Y Liu X Luo S Cheng C. ABIN3 Negatively Regulates Necroptosis-Induced Intestinal Inflammation Through Recruiting A20 and Restricting the Ubiquitination of RIPK3 in Inflammatory Bowel Disease. J Crohns Colitis (2021) 15(1):99–114. doi: 10.1093/ecco-jcc/jjaa131 32599618

[155] Serramito-Gómez I Boada-Romero E Slowicka K Vereecke L Van Loo G Pimentel-Muiños F. The Anti-Inflammatory Protein TNFAIP3/A20 Binds the WD40 Domain of ATG16L1 to Control the Autophagic Response, NFKB/NF-κB Activation and Intestinal Homeostasis. Autophagy (2019) 15(9):1657–9. doi: 10.1080/15548627.2019.1628549 PMC6693451 31184523

[156] Talpin A Kattah M Advincula R Fadrosh D Lynch K LaMere B. A20 in Dendritic Cells Restrains Intestinal Anti-Bacterial Peptide Expression and Preserves Commensal Homeostasis. PloS One (2019) 14(7):e0218999. doi: 10.1371/journal.pone.0218999 31295268 PMC6622485

[157] Pu T Liu W Wu Y Zhao Y. A20 Functions as a Negative Regulator in Macrophage for DSS-Induced Colitis. Int Immunopharmacol (2021) 97:107804. doi: 10.1016/j.intimp.2021.107804 34062371

[158] Lu T Onizawa M Hammer G Turer E Yin Q Damko E. Dimerization and Ubiquitin Mediated Recruitment of A20, a Complex Deubiquitinating Enzyme. Immunity (2013) 38(5):896–905. doi: 10.1016/j.immuni.2013.03.008 23602765 PMC3665706

[159] Martens A Priem D Hoste E Vetters J Rennen S Catrysse L. Two Distinct Ubiquitin-Binding Motifs in A20 Mediate Its Anti-Inflammatory and Cell-Protective Activities. Nat Immunol (2020) 21(4):381–7. doi: 10.1038/s41590-020-0621-9 32205881

[160] Dinallo V Di Fusco D Di Grazia A Laudisi F Troncone E Di Maggio G. The Deubiquitinating Enzyme OTUD5 Sustains Inflammatory Cytokine Response in Inflammatory Bowel Disease. J Crohns Colitis (2021). doi: 10.1093/ecco-jcc/jjab121 34232309

[161] Yang X Sun S. Targeting Signaling Factors for Degradation, an Emerging Mechanism for TRAF Functions. Immunol Rev (2015) 266(1):56–71. doi: 10.1111/imr.12311 26085207 PMC4473799

[162] Rothe M Wong S Henzel W Goeddel D. A Novel Family of Putative Signal Transducers Associated With the Cytoplasmic Domain of the 75 kDa Tumor Necrosis Factor Receptor. Cell (1994) 78(4):681–92. doi: 10.1016/0092-8674(94)90532-0 8069916

[163] Dhillon B Aleithan F Abdul-Sater Z Abdul-Sater A. The Evolving Role of TRAFs in Mediating Inflammatory Responses. Front Immunol (2019) 10:104. doi: 10.3389/fimmu.2019.00104 30778351 PMC6369152

[164] Marzano F Guerrini L Pesole G Sbisà E Tullo A. Emerging Roles of TRIM8 in Health and Disease. Cells (2021) 10(3):561. doi: 10.3390/cells10030561 33807506 PMC7998878

[165] Li Q Lee C Peters L Mastropaolo L Thoeni C Elkadri A. Variants in TRIM22 That Affect NOD2 Signaling Are Associated With Very-Early-Onset Inflammatory Bowel Disease. Gastroenterology (2016) 150(5):1196–207. doi: 10.1053/j.gastro.2016.01.031 PMC4842103 26836588

[166] Zundler S Neurath M. Integrating Immunologic Signaling Networks: The JAK/STAT Pathway in Colitis and Colitis-Associated Cancer. Vaccines (2016) 4(1):5. doi: 10.3390/vaccines4010005 PMC4810057 26938566

[167] Hartjes L Ruland J. CARD9 Signaling in Intestinal Immune Homeostasis and Oncogenesis. Front Immunol (2019) 10:419. doi: 10.3389/fimmu.2019.00419 30906296 PMC6418414

[168] Jukic A Bakiri L Wagner E Tilg H Adolph T. Calprotectin: From Biomarker to Biological Function. Gut (2021) 70(10):1978–88. doi: 10.1136/gutjnl-2021-324855 PMC8458070 34145045

[169] Brost S Koschny R Sykora J Stremmel W Lasitschka F Walczak H. Differential Expression of the TRAIL/TRAIL-Receptor System in Patients With Inflammatory Bowel Disease. Pathol Res Pract (2010) 206(1):43–50. doi: 10.1016/j.prp.2009.09.005 19954896

[170] Akazawa Y Mott J Bronk S Werneburg N Kahraman A Guicciardi M. Death Receptor 5 Internalization Is Required for Lysosomal Permeabilization by TRAIL in Malignant Liver Cell Lines. Gastroenterology (2009) 136(7):2365–76.e1-7. doi: 10.1053/j.gastro.2009.02.071 19272388 PMC2693420

[171] McGovern D Gardet A Törkvist L Goyette P Essers J Taylor K. Genome-Wide Association Identifies Multiple Ulcerative Colitis Susceptibility Loci. Nat Genet (2010) 42(4):332–7. doi: 10.1038/ng.549 PMC3087600 20228799

[172] Rivas M Graham D Sulem P Stevens C Desch A Goyette P. A Protein-Truncating R179X Variant in RNF186 Confers Protection Against Ulcerative Colitis. Nat Commun (2016) 7:12342. doi: 10.1038/ncomms12342 27503255 PMC4980482

[173] Beaudoin M Goyette P Boucher G Lo K Rivas M Stevens C. Deep Resequencing of GWAS Loci Identifies Rare Variants in CARD9, IL23R and RNF186 That Are Associated With Ulcerative Colitis. PloS Genet (2013) 9(9):e1003723. doi: 10.1371/journal.pgen.1003723 24068945 PMC3772057

[174] Zhang H Cui Z Cheng D Du Y Guo X Gao R. RNF186 Regulates EFNB1 (Ephrin B1)-EPHB2-Induced Autophagy in the Colonic Epithelial Cells for the Maintenance of Intestinal Homeostasis. Autophagy (2020) 17(10):1–18. doi: 10.1080/15548627.2020.1851496 PMC8525924 33280498

[175] Bauler L Duckett C O'Riordan M. XIAP Regulates Cytosol-Specific Innate Immunity to Listeria Infection. PloS Pathog (2008) 4(8):e1000142. doi: 10.1371/journal.ppat.1000142 18769721 PMC2516935

[176] Krieg A Correa R Garrison J Le Negrate G Welsh K Huang Z. XIAP Mediates NOD Signaling *via* Interaction With RIP2. Proc Natl Acad Sci USA (2009) 106(34):14524–9. doi: 10.1073/pnas.0907131106 PMC2732880 19667203

[177] Latour S Aguilar C. XIAP Deficiency Syndrome in Humans. Semin Cell Dev Biol (2015) 39:115–23. doi: 10.1016/j.semcdb.2015.01.015 25666262

[178] Aguilar C Latour S. X-Linked Inhibitor of Apoptosis Protein Deficiency: More Than an X-Linked Lymphoproliferative Syndrome. J Clin Immunol (2015) 35(4):331–8. doi: 10.1007/s10875-015-0141-9 25737324

[179] Zeissig Y Petersen B Milutinovic S Bosse E Mayr G Peuker K. XIAP Variants in Male Crohn's Disease. Gut (2015) 64(1):66–76. doi: 10.1136/gutjnl-2013-306520 24572142

[180] Speckmann C Lehmberg K Albert M Damgaard R Fritsch M Gyrd-Hansen M. X-Linked Inhibitor of Apoptosis (XIAP) Deficiency: The Spectrum of Presenting Manifestations Beyond Hemophagocytic Lymphohistiocytosis. Clin Immunol (Orlando Fla) (2013) 149(1):133–41. doi: 10.1016/j.clim.2013.07.004 23973892

[181] Parackova Z Milota T Vrabcova P Smetanova J Svaton M Freiberger T. Novel XIAP Mutation Causing Enhanced Spontaneous Apoptosis and Disturbed NOD2 Signalling in a Patient With Atypical Adult-Onset Crohn's Disease. Cell Death Dis (2020) 11(6):430. doi: 10.1038/s41419-020-2652-4 32514016 PMC7280281

[182] Amininejad L Charloteaux B Theatre E Liefferinckx C Dmitrieva J Hayard P. Analysis of Genes Associated With Monogenic Primary Immunodeficiency Identifies Rare Variants in XIAP in Patients With Crohn's Disease. Gastroenterology (2018) 154(8):2165–77. doi: 10.1053/j.gastro.2018.02.028 29501442

[183] Pedersen J LaCasse E Seidelin J Coskun M Nielsen O. Inhibitors of Apoptosis (IAPs) Regulate Intestinal Immunity and Inflammatory Bowel Disease (IBD) Inflammation. Trends Mol Med (2014) 20(11):652–65. doi: 10.1016/j.molmed.2014.09.006 25282548

[184] Damgaard R Fiil B Speckmann C Yabal M zur Stadt U Bekker-Jensen S. Disease-Causing Mutations in the XIAP BIR2 Domain Impair NOD2-Dependent Immune Signalling. EMBO Mol Med (2013) 5(8):1278–95. doi: 10.1002/emmm.201303090 PMC3944466 23818254

[185] Goncharov T Hedayati S Mulvihill M Izrael-Tomasevic A Zobel K Jeet S. Disruption of XIAP-RIP2 Association Blocks NOD2-Mediated Inflammatory Signaling. Mol Cell (2018) 69(4):551–565.e7. doi: 10.1016/j.molcel.2018.01.016 29452636

[186] Yeh C Bellon M Nicot C. FBXW7: A Critical Tumor Suppressor of Human Cancers. Mol Cancer (2018) 17(1):115. doi: 10.1186/s12943-018-0857-2 30086763 PMC6081812

[187] Yumimoto K Nakayama K. Recent Insight Into the Role of FBXW7 as a Tumor Suppressor. Semin Cancer Biol (2020) 67:1–15. doi: 10.1016/j.semcancer.2020.02.017 32113998

[188] Franke A Balschun T Karlsen T Hedderich J May S Lu T. Replication of Signals From Recent Studies of Crohn's Disease Identifies Previously Unknown Disease Loci for Ulcerative Colitis. Nat Genet (2008) 40(6):713–5. doi: 10.1038/ng.148 18438405

[189] Dhingani N Guo C Pan J Li Q Warner N Jardine S. The E3 Ubiquitin Ligase UBR5 Interacts With TTC7A and May Be Associated With Very Early Onset Inflammatory Bowel Disease. Sci Rep (2020) 10(1):18648. doi: 10.1038/s41598-020-73482-6 33122718 PMC7596066

[190] Jardine S Anderson S Babcock S Leung G Pan J Dhingani N. Drug Screen Identifies Leflunomide for Treatment of Inflammatory Bowel Disease Caused by TTC7A Deficiency. Gastroenterology (2020) 158(4):1000–15. doi: 10.1053/j.gastro.2019.11.019 PMC7062591 31743734

[191] Walden H Rittinger K. RBR Ligase-Mediated Ubiquitin Transfer: A Tale With Many Twists and Turns. Nat Struct Mol Biol (2018) 25(6):440–5. doi: 10.1038/s41594-018-0063-3 29735995

[192] Fiil B Gyrd-Hansen M. The Met1-Linked Ubiquitin Machinery in Inflammation and Infection. Cell Death Differ (2021) 28(2):557–69. doi: 10.1038/s41418-020-00702-x PMC7816137 33473179

[193] Boisson B Laplantine E Dobbs K Cobat A Tarantino N Hazen M. Human HOIP and LUBAC Deficiency Underlies Autoinflammation, Immunodeficiency, Amylopectinosis, and Lymphangiectasia. J Exp Med (2015) 212(6):939–51. doi: 10.1084/jem.20141130 PMC4451137 26008899

[194] Boisson B Laplantine E Prando C Giliani S Israelsson E Xu Z. Immunodeficiency, Autoinflammation and Amylopectinosis in Humans With Inherited HOIL-1 and LUBAC Deficiency. Nat Immunol (2012) 13(12):1178–86. doi: 10.1038/ni.2457 PMC3514453 23104095

[195] MacDuff D Baldridge M Qaqish A Nice T Darbandi A Hartley V. HOIL1 Is Essential for the Induction of Type I and III Interferons by MDA5 and Regulates Persistent Murine Norovirus Infection. J Virol (2018) 92(23):e01368–18. doi: 10.1128/jvi.01368-18 PMC6232484 30209176

[196] Hrdinka M Gyrd-Hansen M. The Met1-Linked Ubiquitin Machinery: Emerging Themes of (De)regulation. Mol Cell (2017) 68(2):265–80. doi: 10.1016/j.molcel.2017.09.001 29053955

[197] Prescott N Lehne B Stone K Lee J Taylor K Knight J. Pooled Sequencing of 531 Genes in Inflammatory Bowel Disease Identifies an Associated Rare Variant in BTNL2 and Implicates Other Immune Related Genes. PloS Genet (2015) 11(2):e1004955. doi: 10.1371/journal.pgen.1004955 25671699 PMC4335459

[198] Sun S. Deubiquitylation and Regulation of the Immune Response. Nat Rev Immunol (2008) 8(7):501–11. doi: 10.1038/nri2337 PMC5763493 18535581

[199] Harhaj E Dixit V. Deubiquitinases in the Regulation of NF-κB Signaling. Cell Res (2011) 21(1):22–39. doi: 10.1038/cr.2010.166 21119682 PMC3075605

[200] Bignell G Warren W Seal S Takahashi M Rapley E Barfoot R. Identification of the Familial Cylindromatosis Tumour-Suppressor Gene. Nat Genet (2000) 25(2):160–5. doi: 10.1038/76006 10835629

[201] Harhaj E Dixit V. Regulation of NF-κB by Deubiquitinases. Immunol Rev (2012) 246(1):107–24. doi: 10.1111/j.1600-065X.2012.01100.x PMC3540820 22435550

[202] Costello C Mah N Häsler R Rosenstiel P Waetzig G Hahn A. Dissection of the Inflammatory Bowel Disease Transcriptome Using Genome-Wide cDNA Microarrays. PloS Med (2005) 2(8):e199. doi: 10.1371/journal.pmed.0020199 16107186 PMC1188246

[203] Cleynen I Vazeille E Artieda M Verspaget H Szczypiorska M Bringer M. Genetic and Microbial Factors Modulating the Ubiquitin Proteasome System in Inflammatory Bowel Disease. Gut (2014) 63(8):1265–74. doi: 10.1136/gutjnl-2012-303205 24092863

[204] Brant S Okou D Simpson C Cutler D Haritunians T Bradfield J. Genome-Wide Association Study Identifies African-Specific Susceptibility Loci in African Americans With Inflammatory Bowel Disease. Gastroenterology (2017) 152(1):206–17.e2. doi: 10.1053/j.gastro.2016.09.032 27693347 PMC5164948

[205] Dufner A Kisser A Niendorf S Basters A Reissig S Schönle S. The Ubiquitin-Specific Protease USP8 Is Critical for the Development and Homeostasis of T Cells. Nat Immunol (2015) 16(9):950–60. doi: 10.1038/ni.3230 26214742

[206] Mevissen T Hospenthal M Geurink P Elliott P Akutsu M Arnaudo N. OTU Deubiquitinases Reveal Mechanisms of Linkage Specificity and Enable Ubiquitin Chain Restriction Analysis. Cell (2013) 154(1):169–84. doi: 10.1016/j.cell.2013.05.046 PMC3705208 23827681

[207] Du J Fu L Sui Y Zhang L. The Function and Regulation of OTU Deubiquitinases. Front Med (2020) 14(5):542–63. doi: 10.1007/s11684-019-0734-4 31884527

[208] Komander D Clague M Urbé S. Breaking the Chains: Structure and Function of the Deubiquitinases. Nat Rev Mol Cell Biol (2009) 10(8):550–63. doi: 10.1038/nrm2731 19626045

[209] Opipari A Hu H Yabkowitz R Dixit V. The A20 Zinc Finger Protein Protects Cells From Tumor Necrosis Factor Cytotoxicity. J Biol Chem (1992) 267(18):12424–7. doi: 10.1016/S0021-9258(18)42292-2 1618749

[210] Catrysse L Vereecke L Beyaert R van Loo G. A20 in Inflammation and Autoimmunity. Trends Immunol (2014) 35(1):22–31. doi: 10.1016/j.it.2013.10.005 24246475

[211] Lork M Verhelst K Beyaert R. CYLD, A20 and OTULIN Deubiquitinases in NF-κB Signaling and Cell Death: So Similar, Yet So Different. Cell Death Differ (2017) 24(7):1172–83. doi: 10.1038/cdd.2017.46 PMC5520167 28362430

[212] Jäättelä M Mouritzen H Elling F Bastholm L. A20 Zinc Finger Protein Inhibits TNF and IL-1 Signaling. J Immunol (Baltimore Md 1950) (1996) 156(3):1166–73. 8557994

[213] Mooney E Sahingur S. The Ubiquitin System and A20: Implications in Health and Disease. J Dental Res (2021) 100(1):10–20. doi: 10.1177/0022034520949486 PMC7755949 32853526

[214] Shembade N Harhaj E. Regulation of NF-κB Signaling by the A20 Deubiquitinase. Cell Mol Immunol (2012) 9(2):123–30. doi: 10.1038/cmi.2011.59 PMC3532050 22343828

[215] Wang K Baldassano R Zhang H Qu H Imielinski M Kugathasan S. Comparative Genetic Analysis of Inflammatory Bowel Disease and Type 1 Diabetes Implicates Multiple Loci With Opposite Effects. Hum Mol Genet (2010) 19(10):2059–67. doi: 10.1093/hmg/ddq078 PMC2860894 20176734

[216] Musone S Taylor K Lu T Nititham J Ferreira R Ortmann W. Multiple Polymorphisms in the TNFAIP3 Region Are Independently Associated With Systemic Lupus Erythematosus. Nat Genet (2008) 40(9):1062–4. doi: 10.1038/ng.202 PMC3897246 19165919

[217] Fung E Smyth D Howson J Cooper J Walker N Stevens H. Analysis of 17 Autoimmune Disease-Associated Variants in Type 1 Diabetes Identifies 6q23/TNFAIP3 as a Susceptibility Locus. Genes Immun (2009) 10(2):188–91. doi: 10.1038/gene.2008.99 19110536

[218] Ciccacci C Latini A Perricone C Conigliaro P Colafrancesco S Ceccarelli F. TNFAIP3 Gene Polymorphisms in Three Common Autoimmune Diseases: Systemic Lupus Erythematosus, Rheumatoid Arthritis, and Primary Sjogren Syndrome-Association With Disease Susceptibility and Clinical Phenotypes in Italian Patients. J Immunol Res (2019) 2019:6728694. doi: 10.1155/2019/6728694 31534975 PMC6732636

[219] Barmada M Brant S Nicolae D Achkar J Panhuysen C Bayless T. A Genome Scan in 260 Inflammatory Bowel Disease-Affected Relative Pairs. Inflamm Bowel Dis (2004) 10(5):513–20. doi: 10.1097/00054725-200409000-00004 15472510

[220] Ma A Malynn B. A20: Linking a Complex Regulator of Ubiquitylation to Immunity and Human Disease. Nat Rev Immunol (2012) 12(11):774–85. doi: 10.1038/nri3313 PMC3582397 23059429

[221] Zheng C Huang Y Ye Z Wang Y Tang Z Lu J. Infantile Onset Intractable Inflammatory Bowel Disease Due to Novel Heterozygous Mutations in TNFAIP3 (A20). Inflamm Bowel Dis (2018) 24(12):2613–20. doi: 10.1093/ibd/izy165 29788367

[222] Lodolce J Kolodziej L Rhee L Kariuki S Franek B McGreal N. African-Derived Genetic Polymorphisms in TNFAIP3 Mediate Risk for Autoimmunity. J Immunol (Baltimore Md 1950) (2010) 184(12):7001–9. doi: 10.4049/jimmunol.1000324 PMC3307531 20483768

[223] Vereecke L Vieira-Silva S Billiet T van Es J Mc Guire C Slowicka K. A20 Controls Intestinal Homeostasis Through Cell-Specific Activities. Nat Commun (2014) 5:5103. doi: 10.1038/ncomms6103 25267258

[224] Bank S Andersen P Burisch J Pedersen N Roug S Galsgaard J. Associations Between Functional Polymorphisms in the Nfκb Signaling Pathway and Response to Anti-TNF Treatment in Danish Patients With Inflammatory Bowel Disease. Pharmacogenomics J (2014) 14(6):526–34. doi: 10.1038/tpj.2014.19 24776844

[225] Lu D Song J Sun Y Qi F Liu L Jin Y. Mutations of Deubiquitinase OTUD1 Are Associated With Autoimmune Disorders. J Autoimmun (2018) 94:156–65. doi: 10.1016/j.jaut.2018.07.019 30100102

[226] Yang S Hong M Zhao W Jung Y Tayebi N Ye B. Genome-Wide Association Study of Ulcerative Colitis in Koreans Suggests Extensive Overlapping of Genetic Susceptibility With Caucasians. Inflamm Bowel Dis (2013) 19(5):954–66. doi: 10.1097/MIB.0b013e3182802ab6 23511034

